# National Policies to Prevent and Manage Cervical Cancer in East African Countries: A Policy Mapping Analysis

**DOI:** 10.3390/cancers12061520

**Published:** 2020-06-10

**Authors:** Diana Wangeshi Njuguna, Nour Mahrouseh, Dede Onisoyonivosekume, Orsolya Varga

**Affiliations:** 1 Department of Preventive Medicine, Faculty of Public Health, University of Debrecen, Kassai Str, 4028 Debrecen, Hungary; nourmahrouseh93@gmail.com (N.M.); varga.orsolya@sph.unideb.hu (O.V.); 2 Faculty of Medicine, University of Debrecen, 4032 Debrecen, Hungary; dede.oyvke@gmail.com

**Keywords:** policy mapping, policy analysis, cervix cancer, East African countries, HPV vaccination

## Abstract

Policy mapping is used to provide evidence on effective interventions and highlight the necessary refinements of health policies. The goal of this work is to carry out legal mapping to identify and assess health policies for the prevention and management of cervical cancer in East African countries. Cervical cancer, as a largely preventable disease, is the cause of most cancer deaths among women in East African countries. Legal documents were searched uniformly from government websites, national cancer institute sites, and international and national legal databases, then the data were analyzed using the Nvivo12 software package. The sample of 24 documents includes policies, plans, guidelines, acts, and strategies from 12 East African countries. The emerging themes were screening, prevention, diagnosis, treatment, challenges, and mitigation efforts. A few binding policies, a significant discrepancy to international standards in at least four countries, patchy screening registries, and a lack of prophylactic vaccination against human papillomavirus incorporation into national immunization schedules are the main findings. This paper underlies the role of law in health and the need for transparent legal and regulatory tools to achieve a further reduction in cervical cancer mortality in East African countries.

## 1. Introduction

### 1.1. Burden of Cervical Cancer in East African Countries

In 2018, cervical cancer was the fourth most common cancer worldwide in women, contributing to 6.9% of the total number of new cases [1]. Globally, among the 20 countries with the highest incidence of cervical cancer, 16 are African countries. From the total number of cancer deaths among women in Sub-Saharan Africa (SSA), 19.3% are attributed to cervical cancer [2]. In 2018, according to Global Cancer Observatory data for East Africa, cervical cancer had the highest incidence among women, with 52,633 new cases and 37,017 deaths [3].

Cancer of the cervix is mainly attributed to human papillomavirus (HPV) types 16 and 18. According to a recent report by the HPV Information Centre, the most prevalent oncogenic types among women in the East Africa are HPV 16, followed by HPV 18 and HPV 52 [4]. However, recently, mainly cross-sectional studies have reported on the diversity of HPV types within and across countries, affecting the efficacy of HPV vaccinations, e.g., the diversity of prevalent HPV types in Ethiopia [5]. Other risk factors that have been associated with cervical cancer include HIV/AIDS, family history, lifestyle, and oral contraceptive use [6,7]. Vaccination against the major subtypes of HPV, i.e., HPV 16 and 18, will reduce the burden of cervical cancer in the future among women in SSA [8], although none of the three vaccines in the market are able to give full protection. However, they are beneficial to some extent (depending on the local prevalence of the given HPV type) [9]. Cancer detection in developing regions typically occurs late, mainly due to inadequate primary health care and treatment interventions [10]. Developed countries have well-structured healthcare systems, the benefits of which are not attainable in developing countries due to a lack of resources. Developed countries offer access to affordable and high-quality cancer care for all. The issues of delayed results, poor screening procedures, lack of follow-up, and late diagnosis have highly contributed to cancer being a public health burden in low- and middle-income countries [11]. However, cancer detection and treatment may also occur late due to a lack of individual awareness. A qualitative study on the beliefs, perceptions, and health seeking behaviors in relation to cervical cancer in Uganda mentioned late diagnosis as a barrier to the reduction of cervical cancer [12].

### 1.2. Public Health Policies Are Effective in Reducing Disease Burden

Public health cannot be effective without laws, and countries cannot achieve their goals unless the necessary legal and regulatory tools have been developed and implemented. In recent years, policy mapping has emerged as an approach to provide transparency in health laws [13,14].

The most common types of legal instruments included in legal mapping studies are laws, regulations, and policies. Laws exist to govern behavior, while regulation, in the public health domain, is the use of instruments of various types to implement these socioeconomic policies or laws [15]. Law is essential to achieve health goals concerning population health, e.g., reducing the burden of cancer by reducing its prevalence in a country. An act, as a type of law, is a specific term used for a rule passed by parliament. Legal regulation is used to establish public health agencies, delegate their core functions, allocate funding, and also to set the limits within their actions to protect the rights of individuals. The phrase “policy” is often used broadly to include laws and regulations intended to accomplish certain goals. In a narrower sense, policies often differ from laws in the way that they are enforced and the consequences of noncompliance [16].

Some agencies also publish guidelines or other non-binding policy statements which further clarify how an agency understands and implements existing laws and regulations. Guidelines and other policy statements describe suggested or recommended actions. Guidelines and policy statements do not provide mandatory requirements, unless they are incorporated into a regulation or are mandated under the terms and conditions of an agreement, such as a funding agreement [17]. Strategy has numerous interlinks with the term policy. Strategy, as a non-binding action, is the set of decisions concerning a specific scope of interest that the government intends to implement according to a plan. Discussion emphasizes the foresight to divide government actions into tangible specific programs to achieve the ultimate goal [18].

While national cancer control legislations (hereinafter referred to as policies) are imperative to set up [19], they are a commitment by the government, whose aim is to provide a foundation for the implementation and maintenance of cancer control programs [20], and evidence has shown that relevant health care policies provide a boost in improving clinical outcomes [21]. Cancer policies give cancer visibility that results in tangible gains in the management of cancer. Some types of cancer (e.g., lung cancer) incidences have declined due to health policies [22].

According to the American Cancer Society, screenings for cervical cancers have been proven to reduce mortality from these cancers by detecting them earlier, where they are more likely to be treated successfully. Indisputably, screening for cervical cancer reduces morbidity and mortality by 30% within three years of the implementation of screening results [23]. Evidence has shown that there are constraints in the health systems in Sub-Saharan Africa (SSA) that enhance hitches in the actual implementation of cervical cancer interventions [24]. In her article on human rights meeting sound health policy, Ginsburg highlights that the worth of effective cervical cancer control policies in of itself is a basis for the empowerment of women [25].

### 1.3. Guidelines on Cervical Cancer

Cervical cancer screening is recommended for women to detect precancerous changes in the cervix [26]. The guidelines for the prevention and early detection of cervical cancer highlight the age appropriate high-quality cytology testing known as a Papanicolaou test (Pap smear). It is a primary screening test for the early detection of precancerous cancers of the cervix [7,27].

Comprehensive cancer control programs have four important components, which are prevention, early detection, diagnosis, and treatment and palliative care [28]. Cervical cancer control programs are designed to aid in the reduction of the incidence of cancer and improve quality of life [7]. The strategies for cervical cancer control and prevention are early detection by effective screening for HPV, vaccination against HPV, Pap smear testing, and the treatment of precancerous lesions [29]. For low resource settings, the screen and treat approach for cervical cancer control has been successful; this is achieved by visual inspection with acetic acid (VIA) and visual inspection with Lugol’s iodine (VILI), which has a high sensitivity of 100% to detect any dysplasia in the cervix, which is then followed by treatment using cryotherapy [30,31].

While several national and professional guidelines exist, the comprehensive cervical cancer control guide to essential practice by the World Health Organization (WHO) gives a broad vision of what a comprehensive approach to cervical cancer prevention and control means. This international guide gives evidence-based information on what works in cervical cancer prevention and control programs to national-level decision-makers, which may be of use as a basis for updating their own policies, including guidelines and protocols [7].

### 1.4. The Aim of the Study

Our aims are as follows: (1) identification and content analysis of policies for the prevention and management of cancer of the cervix of East African countries, (2) and determination of their compliance with the comprehensive cervical cancer control guide to essential practice laid out by the WHO.

## 2. Methods

### 2.1. Study Area and Design

Systematic document searching and analysis of health policies on cervical cancer prevention and/or management from East African countries was carried out in Burundi (BI), Comoros (KM), Democratic Republic of Congo (DRC), Djibouti (DJ), Eritrea (ER), Ethiopia (ET), Kenya (KE), Madagascar (MG), Rwanda (RW), Somalia (SO), Uganda (UG), and Tanzania (TZ) [32,33].

### 2.2. Searching

The search for data used a systematic approach, where data were searched from national/state government websites, health ministry websites, national cancer institute sites, international and national legal databases, e.g., LexisNexis and Westlaw, the African legal information institute, the international cancer control partnership portal, and the WHO non-communicable disease document repository. A detailed list of databases and websites is available in Supplementary 1. The search was conducted from February to June in 2019. The search terms for the international databases included the country name and cervical cancer or cancer depending on the filtering option of the website. The search terms of national databases were cervical cancer or cancer depending on the filtering option of the website, without any restrictions.

### 2.3. Screening

The following inclusion criteria was used: national legal documents that were published under health-related ministries, governmental institutions, national cancer institutes, or parliaments. Any legal instruments (e.g., guidelines, strategies, plans, policies, regulations, or acts of parliament) were eligible for inclusion when they described aspects of the prevention, management, and control of cervical cancer. Legal documents were excluded if there was no information on cervical cancer available in the whole document, e.g., national non-communicable disease plans that had generalized data on cancer as opposed to highlighting the specific issues on cervical cancer. Non-legal texts, e.g., articles or grey literature, were also excluded. Screening of the data and the selection process for each country are presented by 12 PRISMA flow charts (see Figures S1–S12).

### 2.4. Data Coding and Analysis

The data found were in different languages; mainly English, Kiswahili, and French. Data in Kiswahili and French was translated. The data collected were uploaded into the QRS Nvivo 12 Plus software package for content analysis of unstructured qualitative data. Related information was selected and put in nodes. Coding of the data was developed by two researchers (DWN, OV) according to the WHO national cancer control programs, policies, and managerial guidelines [34]. The data categories were coded in two stages, with the second stage taking a more refined approach.

### 2.5. Quality Appraisal

The quality appraisal (internal validity) consisted of an assessment tool and a response scale. The validated tool provides relationships between policy determinants and expected outcomes [35,36]. The tool was used to evaluate each policy document based on 7 domains (background, goals, resource considerations, monitoring and evaluation, public opportunity, obligations, and potential for public health impact). These domains represent the essential data that a policy should be evaluated upon. The response scale was on a 3-point nominal scale that ranged from 0 to 2 (0 represented not fulfilled/weak, 1 represented room for improvement, and 2 represented fulfilled). Based on these domains, each policy document was assessed and given scores for each domain according to the data provided in the text (See Table S1). This was systematically carried out by two independent reviewers (DWN, NM). Contradictions were resolved by open discussion. Inter-rater reliability for each document and each domain was assessed using Cohen’s kappa (k) inter-rater reliability statistic, using SPSS statistics for Windows, version 25.0. The categorization was based on the thresholds recommended by Fleiss [37,38].

## 3. Results

### 3.1. Description of the Policies

The sample included policies, plans, guidelines, acts and strategic documents from Kenya, Uganda, Tanzania, Burundi, Rwanda, Eritrea, Ethiopia, Madagascar, the DRC, and Comoros. No relevant policies were identified from Somalia and Djibouti. A breakdown of the 24 included documents is provided in Table 1 below. Out of the 24 legal documents, only 4 acts were identified that are legally binding.

### 3.2. Quality Appraisal

The mean of the sum of scores for internal validity was 7.63 (95% Confidence Interval (CI): 5.85–9.40). The scores ranged from 0 to 13, shown in Table S1 of Supplementary Document 3. The inter-rater reliability of the sum of scores was determined using the intraclass correlation coefficient (ICC), which was high (excellent), with a value of 0.947 (95% CI: 0.876–0.977). The policy document with the highest score was the Kenya National Cancer Prevention Strategy 2017–2022 (score 13) and the domain with the highest score was public opportunity, with a mean of 1.33 (95% CI: 1.01–1.65), while the lowest was resource considerations, with a mean of 0.79 (95% CI: 0.51–1.07). The inter-rater reliability was tested for all the retrieved documents. Cohen’s kappa (k) inter-rater reliability statistic, using the SPSS software package, was used for each domain. These results (see Table 2) indicate the level of agreement/disagreement of the two raters for the policies that have been provided. The scores for each domain and each document between the two raters were discussed and disagreements were solved with open discussion, presented in Table S3 of the Supplementary Materials.

### 3.3. Overview of Themes

The emerging parent nodes were global burden, justification for regulation, epidemiology, risk factors, validity period, edition year, target group, screening, treatment, diagnosis, and prevention. The nodes were grouped into themes, which were screening, prevention, diagnosis and treatment, and challenges and solutions. These nodes are presented in more detail below.

### 3.4. Screening

The screening theme covers the aspects that entail the systematic application of methods of identifying women with suspicious lesions that are suggestive of cervical cancer, who then need referral for further investigation [28]. Under this theme, the goal of the cervical screening and the provided methods are described below. This theme covers 8 child nodes with 96 references (the number of relevant text parts that were coded at a node) from 15 files. The goal of the screening, as expressed by the Ethiopian National Cancer Control Plan, is that “some cancers like breast and cervical cancer can benefit from early screening and detection and from treating the disease before it grows into an advanced stage”. The following screening methods were included in the policies, explaining their advantages and limitations: colposcopy, cytology with pap smear test, early detection, HPV DNA tests, single visit see-and-treat approach (SVA), VIA combined with cryotherapy, VIA, and VILI. VIA was discussed in 12 files with 25 references, while VILI was mentioned in 10 files with 17 references. These two methods, VIA and VILI, were the most commonly discussed methods in the legal documents in the countries, illustrated by Table 3 below. Cytology with a Pap smear test was also severally cited, for example, the Kenya National Cervical Cancer Prevention Plan highlighted it as a limitation, where the “Pap smear is the most commonly used method for cervical cancer screening, but its availability is limited to the urban areas. The long waiting time between screening and obtaining results leads to many clients getting lost in the follow-up period” [58].

### 3.5. Prevention

The prevention theme was geared towards minimizing exposure to the causes of cancer and reducing individual susceptibility to the effects of these causes, thus offering the ultimate potential public health control of cancer which is also cost effective [34]. This theme was grouped by 11 nodes with 75 references from 13 files. The nodes in this category were abstinence from sexual exposure, adoption of a healthy lifestyle, advocacy, being mutually faithful, capacity building, consistent condom use, creating awareness of cancer and reproductive health education, promoting male circumcision, the discouragement of tobacco use, VIA combined with cryotherapy, and HPV vaccination. Creating awareness of cancer and reproductive health education received the vast majority of attention from legislators, as shown in Table 4.

### 3.6. Diagnosis and Treatment

Diagnosis and treatment are part of the secondary prevention component for cancer prevention, to decrease incidence and lower the prevalence of cervical cancer and the associated mortality by intercepting the progress from pre-cancer to invasive cancer [7]. This theme highlighted the procedures undertaken to elucidate the certainty of cervical cancer and the interventions to reduce susceptibility. Four nodes emerged in the diagnosis theme, with 27 references from 10 files and 7 nodes for the treatment theme, with 104 references from 13 files. The 4 nodes in the diagnosis theme were colposcopy, cervical biopsy, cervical cytology (Pap smear), and staging, found from the DRC, Madagascar, Kenya, Uganda, and Ethiopia. Cervical biopsies, colposcopies, and endo-cervical curettages were the most commonly mentioned diagnostic tests for cervical pre-cancer [51]. Cervical biopsies (*n* = 6) were the most common, followed by colposcopies (*n* = 5). The Ministry of Health in Kenya highlighted that the confirmation of a diagnosis is an essential step, thereby recommending colposcopies and cervical biopsies [59]. The nodes for treatment were chemotherapy (*n* = 9), cone biopsies (*n* = 8), cryotherapy (*n* = 9), curative surgery (*n* = 12), hospice care (*n* = 8), radiotherapy (*n* = 13), and loop electrosurgical excision procedures (*n* = 7), from Tanzania, Uganda, Kenya, Madagascar, Ethiopia, and the DRC.

### 3.7. Challenges and Solutions Articulated in the Policies

There were challenges which were identified as hindrances to curbing the burden of cancer, due to several obstacles from 100 references from 17 files covering 11 nodes. Solutions were the approaches or measures that have been suggested to curtail the burden of cancer. There were 77 references from 17 files incorporating 8 nodes for this.

The challenge theme emerged from the late stage detection (*n* = 15), high cost of treatment (*n* = 9), chronic diseases (*n* = 3), lack of information (*n* = 7), poor infrastructure (*n* = 10), long waiting time (*n* = 1), scant records (*n* = 6), low screening rates (*n* = 2), socio-cultural issues (*n* = 3), poor funding (*n* = 3), uncoordinated screening services (*n* = 2), lack of capacity (*n* = 8), and lack of regulation (*n* = 1) nodes.

Late stage or advanced stage at detection was cited by most countries as the biggest challenge faced by East African countries. Poor infrastructure for the prevention and control of cervical cancer was also highly mentioned, referring to a lack of capacity and uncoordinated screening services. A high cost of treatment and lack of information were highly cited from Kenya, Madagascar, the DRC, Uganda, and Ethiopia. As was explained by a policy from Madagascar, the stakes of the fight against cancer in Madagascar are summed up by a lack of information to the general public on the issue, the physical and financial inaccessibility of support services that are concentrated in the capital, a reference system against faulty references, and helpless patients who rely heavily on alternative medicines [46].

The solutions theme included the nodes of initiation of prevention, the screening and management of reproductive organ cancer at all levels (*n* = 6), the development and implementation of national cervical cancer prevention and control programs (*n* = 6), the establishment of appropriate surveillance strategies and the integration of other national policy documents (*n* = 6), and the training of health officers for screening and treatment and consequently deploying them (*n* = 6). These were the highly cited efforts to mitigate cancer in most of the East African countries. Additional and marginalized nodes included the scaling of screening and treatment of cervical cancer (*n* = 5), the coordination of activities and resource mobilization (*n* = 4), cancer education and community mobilization strategies (*n* = 2), and the continuation of previous strategies (*n* = 1).

### 3.8. Comparing National Policies of East African Countries to the WHO Comprehensive Cervical Cancer Control Guide to Essential Practice

The main objective of cervical cancer national policies is to decrease the incidence and prevalence of cervical cancer and any mortality associated with cervical cancer. To achieve this objective, countries must develop national policies, e.g., guidelines and protocols for cervical cancer prevention and control, based on evidence. According to the WHO comprehensive cervical cancer control guide to essential practice [7], there are essential elements to be addressed in national guidelines and protocols for cervical cancer prevention and control, including HPV vaccination, screening, treatment for precancerous lesions, treatment for invasive cancer, and a functioning referral system. Our findings, as presented in Table 5, show that the majority (*n* = 8) of the East African countries covering Kenya, Uganda, Tanzania, Madagascar, Rwanda, Ethiopia, Burundi, and the DRC had adhered to the requirements. However, some countries are yet to include HPV vaccination into their national immunization schedules. Out of the 12 countries, Somalia, Eritrea, Comoros, and Djibouti did not have any information on HPV vaccination. Regular screening must be linked to treatment, and the majority (*n* = 9) of countries had screening activities. Slightly more than half (*n* = 7) of the countries regulate the procedures for the treatment for precancerous lesions, while only six countries had mentioned the treatment of invasive cancer. An effective referral system prevents cases of loss to follow-up. Our findings highlight that only six countries cited referral mechanisms for patients with cancer of the cervix.

## 4. Discussion

East African countries have made tremendous efforts in terms of coming up with policies to prevent and control the growing burden of cervical cancer since 1996. The majority (83%) of the countries have policies related to cervical cancer and have touched on components of its control, as recommended by the WHO, i.e., prevention, early detection, treatment, and palliative care [28]. However, the identified policies were mainly without executive rules, indicating an unclear and patchy regulatory situation.

The prevailing practices in the control of cervical cancer in most policies highlight colposcopies, cytology with a Pap smear test, HPV DNA tests, SVA, VIA, and VILI. The WHO Comprehensive cervical cancer control guide to essential practice recommends these practices for the screening and treatment of precancerous cases, due to the evidence of their capacity to reduce the incidence of cervical cancer [7,63]. This finding is also in line with literature recommending the use of VIA and VILI as simple screening methods under limited resources [30,64,65]. VIA and VILI can be performed at low level hospitals (i.e., hospitals providing a narrow range of services), since they are cheap, non-invasive, and the results are instant. Linkage between screening and treatment also contributes to wide use in low resource settings. For women who are found to be eligible or have precancerous lesions, treatment is carried out via cryotherapy immediately [66,67]. These techniques (VIA and VILI) are part of the single visit see-and-treat approach, as they rely on visual inspection and are scalable for women [68].

Screening by HPV DNA tests is costly, as cited by several studies carried out in Africa [69,70]. HPV DNA testing is recommended due to its sensitivity, which is better than what is achieved by cytology [71]. The use of HPV DNA testing is limited in low resource settings [72], due to the expensive laboratory infrastructure required to process the results. Catarino et al. evaluated the challenges in implementing screening programs in low- and middle-income countries and recommended the introduction of point-of-care HPV testing to make screening more feasible and reduce the infrastructural requirements, hence prompting results and interventions [67].

Cervical cancer is a potential preventable non-communicable disease, hence, efforts to prevent it should be highly emphasized [73] in countries where cervical cancer is a public health burden. The WHO recommends the inclusion of HPV vaccination into national immunization programs, hence promoting the prevention of cervical cancer [74]. HPV vaccination programs have shown a significant reduction in cervical cancer burden. This has been illustrated by a study conducted in Australia, where 11- to 13-year-old girls were vaccinated. Because of the study’s success, the program was later expanded to vaccinate boys aged 11 to 12 years [75]. There are only two countries (Rwanda and Uganda) which have national vaccination programs and five (Burundi, Ethiopia, Kenya, Madagascar, and Tanzania) with pilot programs that are partially or totally supported by the Global Alliance for Vaccines and Immunization (GAVI) [4].

For the execution of cost-effective cancer prevention strategies, capacity building is crucial. Most policies emphasized this intervention by highlighting measures like conducting collaborative seminars and workshops, pursuing medical education, training health practitioners, continuous updates, and conducting research [76,77]. Increasing cancer awareness, sensitization about cancer and social mobilization and lobbying were approaches embraced by most countries. This is an essential component in cancer control programs [78,79]. There are initiatives based on community involvement and participation and also monitoring systems to enforce policies, including screening and vaccination programs. Creating public awareness helps improve general knowledge and perception. The policies highlighted creating awareness of cervical cancer and reproductive health as a key preventive measure, more specifically, age appropriate sexual health education among young girls [80]. However, this has been severally underscored in the literature [81]. According to the WHO, the screening of women is the first step towards identifying women in the population who have cervical cancer, then further investigating to confirm the diagnosis or disregard the case [20].

The treatment of cervical cancer is mainly carried out via chemotherapy, radiotherapy, and curative surgery, depending on the stage of the cervical cancer. There is a disparity for treatment between developed countries and Africa, due to differences in resource availability. While developed countries have readily available technology and personnel to implement the most appropriate treatment modalities for cancer of the cervix, countries in Africa are grappling with vaccination and screening, and the countries are yet to advance to diagnosis and treatment [82]. Anorlu (2008) concluded that in Africa, precancerous lesions are treated by curative surgery (hysterectomy) and cone biopsies, with radiation, surgery, and palliative care for invasive cervical cancer [83], which corresponds with our findings.

### 4.1. Challenges and Solutions

Cervical cancers in SSA are detected at a late stage, predominantly due to a lack of information about cervical cancer and a scarcity of prevention services [63]. In this regard, late-stage disease is associated with low survival rates after surgery or radiotherapy [84]. Inaccessibility to health care services due to poor infrastructure [85] and a lack of drugs and screening facilities, coupled with a lack of human resources for health, is the basis for low screening rates [86]. Low screening rates were associated with a lack of information about cervical cancer [87]. The long waiting time to receive cytology results leads to loss of follow-up, which is a contributing factor to advanced stage diagnosis [88]. Poor funding, as cited by many East African countries, is congruent with several studies conducted in Africa on a lack of funding as a barrier to the control and prevention of cancer [63,89]. Populations of East African countries are not covered by a social healthcare system, where there is no universal access to most healthcare services, for example, HPV vaccination. The challenge is that countries are yet to include HPV vaccination in their national immunization schedules, coupled with constraints to implementation and sustainability attributed to poor funding, due to incremental system costs that come with the availability and delivery of vaccines to the health centers and proper handling of the vaccines [79,80,90,91]. At this point, health care costs are covered by the respective governments, with support from GAVI and international pharmaceutical institutions. Currently, 7 out of 12 countries are providing these services at a free cost for girls [92].

The solutions vastly cited by East African countries include the initiation of prevention, screening, and management of reproductive organ cancers at all levels. According to Maine et al. (2011), the development and implementation of national cervical cancer prevention and control programs and establishing appropriate surveillance strategies and integrating other national policy documents are steps towards reducing the burden of cancer [29]. Capacity building by training health officers on screening and treatment and deploying them is key to scaling human resources for health [67].

Advocacy is probably the key in reversing the cancer burden, by pushing for budget allocation and the mobilization of resources and forming partnerships with key stakeholders to be able to respond passably to the cancer crisis [93]. Capacity building to strengthen health systems is essential. All the countries studied emphasized that capacity building is critical in controlling and preventing cancer. This is well achieved by training new and existing health personnel who will competently carry out the activities related to the cancer control programs [93].

### 4.2. Need for Evidenced-Based Health Policies on Cervix Cancer Prevention

Public health policy should provide evidence-based solutions to cervical cancer. Our major findings highlight the low number of binding and old policies and the discrepancy to international guides. Additionally, very limited data were found reporting morbidity and mortality as evidence for policy making. A strengthened surveillance system and better knowledge of policy makers on how to incorporate the evidence to their policy agenda to inform budgetary priorities would reduce the harmful impact of the discordance between evidence and policy [94].

The majority of East African countries have not established screening registries that incorporate individuals’ screening data with cancer registry, as is required [95]. The registries aid in the keeping of records, which are an essential tool for monitoring and evaluation. In contrast, the scarcity of data in East African countries leads to poor or no reliable data for assessing the cancer burden in the population. The establishment of screening registries and the linkage of individual screening data with a cancer registry will contribute to reducing disparities in cancer outcomes [96]. Detailed registries are vital to provide specific population needs, and hence, in order to prioritize the allocation of resources [97].

There are financial, logistical, and sociocultural constraints that are hindrances that have contributed to a poor disclosure of policies and the implementation of screening programs, contrary to high income countries [98].

### 4.3. Implications

There is an urgent need for policy makers of health in East African countries to increase legal transparency and prioritize the development and implementation of cancer policies, whereby past strategies have not worked, but have been repeated, creating a wide gap between what is needed and what needs to be practiced [99]. Binding and non-binding policies are important tools of public health. The severe gaps in national policies mirror the weaknesses of the public health field. Our major findings show few and outdated policies on cervical cancer, highlighting the need for scientifically sound laws and their implementation by executive rules.

## 5. Conclusions

Evidence-based policy mapping plays an important role in providing insight on the management of disease, thus, in this way, governments are able to make informed decisions for health [100]. Legal mapping for cancer is especially important, because it provides effective insight to stakeholders in policymaking by the effective implementation of cancer policies. Reviewing outdated cervical cancer policies to update information with evidence-based best practices is urgently required [63], as well as ensuring transparent access to legal information in these countries. East African countries should put concentrated effort into cervical cancer policies, e.g., pooling funds to support cervical cancer control programs and making HPV vaccines available.

## Figures and Tables

**Table 1:** List of policy documents by counties, title and year of publication.

Country	Title of Policy	Type	Year of Publication	Reference
Burundi	National Health policy	Policy	2016	[39]
Democratic Republic of Congo	National strategy to combat the cancer of the uterine neck and breast	Strategy	Not available	[40]
Eritrea	Health Sector Strategic Development plan	Strategy	2016	[41]
Ethiopia	Guideline for cervical cancer prevention and control	Guideline	2015	[42]
	National Cancer Control Plan	Plan	2015	[43]
Comoros	Reproductive health policy	Policy	2002	[44]
Madagascar	National Strategic Plan to fight against cervical cancer	Strategy	2016	[45]
	National cancer policy	Policy	2010	[46]
	Cervical cancer screening guide	Guideline	Not available	[47]
Uganda	Uganda Cancer Society Strategic Plan	Strategy	2016	[48]
	Cervical Cancer strategic Plan	Strategy	2010	[49]
	Cancer Institute Act	Act	2017	[50]
	Uganda cancer institute treatment guidelines	Guideline	2017	[51]
Tanzania	The ocean road cancer institute	Act	1996	[52]
	National cancer strategic plan	Strategy	2013	[53]
	Cervical cancer strategic plan	Strategy	2011	[54]
Rwanda	Ministry of Health strategic plan	Strategy	2018	[55]
Kenya	Cancer prevention and Control (Amendment Bill)	Act	2016	[56]
	Cancer prevention and Control Act	Act	2012	[57]
	National Cervical Cancer Prevention Plan	Plan	2012	[58]
	National Guidelines for Prevention and management of Cervical Breast and Prostate Cancers	Guideline	2012	[59]
	National Cancer Treatment Guidelines	Guideline	2013	[60]
	National Cancer Control strategy	Strategy	2011	[61]
	National Cancer Control strategy	Strategy	2017	[62]

Legend: 24 legal instruments from 10 countries, with their legislative type i.e., policies, plans, guidelines, acts and strategic documents.

**Table 2:** Inter-rater reliability for the domains of internal validity.

Domain	No Agreement *k* (≤0)	Slight *k* (0.01–0.20)	Fair *k* (0.21–0.40)	Moderate *k* (0.41–0.60)	Substantial *k* (0.61–0.80)	Excellent *k* (>0.75)	Almost Perfect Agreement *k* (0.8–1.00)
Background and case for change					0.645		
Goals					0.670		
Resources					0.714		
Monitoring and evaluation				0.515			
Public opportunities				0.467			
Obligations			0.263				
Potential for public impact			0.262				

Legend: Inter-rater reliability was expressed by Cohen’s kappa (k). Calculation is based on the internal validity scores, available in Tables S2 and S3.

**Table 3:** Coding presence for the different modalities of screening among the East African countries.

Country	Cytology Using Pap Smear Test	Early Screening and Detection	HPV DNA Tests	SVA	VIA Combined with Cryotherapy	VIA	VILI
Burundi	No	No	No	No	No	No	No
Comoros	No	No	No	No	No	No	No
DRC	No	No	Yes	No	No	Yes	Yes
Eritrea	No	No	No	No	No	No	No
Kenya	No	Yes	Yes	Yes	Yes	Yes	Yes
Ethiopia	No	Yes	Yes	No	Yes	Yes	Yes
Madagascar	Yes	Yes	Yes	No	Yes	Yes	Yes
Rwanda	No	No	No	No	No	Yes	No
Tanzania	No	Yes	Yes	Yes	Yes	Yes	Yes
Uganda	No	Yes	Yes	Yes	No	Yes	Yes

Legend: Kenya, Ethiopia, Madagascar, Tanzania and Uganda have most screening modalities in the legal instruments, as compared to the rest of the countries. DRC—Democratic Republic of Congo, HPV DNA—Human papillomavirus DNA test, SVA—Single visit see-treat-approach, VIA—visual inspection with acetic acid, VILI—visual inspection with Lugol’s iodine.

**Table 4:** Prevention nodes against the East African countries.

Prevention Nodes	BI	KM	DRC	ER	ET	KE	MG	RW	TZ	UG
Abstinence from sexual exposure	0	0	0	0	1	2	0	0	0	0
Adoption of healthy lifestyle	0	0	0	0	0	0	6	0	0	0
Advocacy *	0	0	3	0	2	4	1	0	1	3
Being mutually faithful	0	0	0	0	1	2	0	0	0	0
Capacity building	0	0	0	0	1	0	0	0	1	0
Consistent condom use	0	0	0	0	1	2	0	0	1	1
Creating awareness on cancer and reproductive health education *	0	0	3	0	3	6	3	0	2	8
Discouragement of tobacco use	0	0	1	0	0	1	1	0	1	0
HPV vaccination **	0	0	2	0	1	5	3	0	3	2
Promote male circumcision	0	0	0	0	0	2	0	0	0	0
VIA combined with cryotherapy	0	0	0	0	1	0	0	0	0	0

Legend: * Highlights the main intervention plans to reduce cervical cancer in East African countries. ** Prophylactic vaccination against HPV is yet to be included in the national immunization schedules. Burundi (BI), Comoros (KM), Democratic republic of Congo (DRC), Eritrea (ER), Ethiopia (ET), Kenya (KE), Madagascar (MG), Rwanda (RW), Uganda (UG) and Tanzania (TZ).

**Table 5:** Adherence to the WHO recommendations by countries.

Countries	HPV Vaccination	Screening	Treatment for Precancerous Lesions	Treatment for Invasive Cancer	Referral System
Kenya	Yes	Yes	Yes	Yes	Yes
Uganda	Yes	Yes	Yes	Yes	Yes
Tanzania	Yes	Yes	Yes	Yes	Yes
Madagascar	Yes	Yes	Yes	Yes	Yes
Somalia	No data	No data	No data	No data	No data
Rwanda	Yes	Yes	Yes	Yes	Yes
Eritrea	No	No	No	No	No
Ethiopia	Yes	Yes	Yes	Yes	Yes
Burundi	Yes	Yes	No	No	No
Comoros	No	Yes	No	No	No
Djibouti	No data	No data	No data	No data	No data
DRC	Yes	Yes	Yes	No	No

Legend: This table demonstrates how the cervical cancer legal documents from the different countries had adhered to five essential requirements by the WHO Comprehensive Cervical Cancer Control guide to essential practice [7]. Yes means that the legal documents in a country fulfilled the essential elements, No means lack of information in the national legal documents, while no data means lack of any relevant legal documents from a country.

## Supplementary Materials

The following are available online at https://www.mdpi.com/2072-6694/12/6/1520/s1, Figure S1: Selection of legal documents for Burundi, Figure S2: Comoros, Figure S3: Djibouti, Figure S4: Democratic Republic of Congo, Figure S5: Eritrea, Figure S6: Ethiopia, Figure S7: Kenya, Figure S8: Madagascar, Figure S9: Rwanda, Figure S10: Somalia, Figure S11: Tanzania, Figure S12: Uganda, Table S1: Internal validity assessment for the included legal documents, by domains, Table S2: Internal validity assessment for the included legal documents, by domains, by Reviewer 1, Table S3: Internal validity assessment for the included legal documents, by domains, by Reviewer 2, Supplementary data: List of Databases and Websites.
