# Supplementary material for: National Policies to Prevent and Manage Cervical Cancer in East African Countries: A Policy Mapping Analysis
Source: Cancers (Basel). 2020 Jun 10;12(6):1520. doi: 10.3390/cancers12061520 (PMC7352307; doi:10.3390/cancers12061520)
Supplement: Supplementary file 1 [file cancers-12-01520-s001.pdf]

Supplementary Materials: National Policies to Prevent and Manage Cervical Cancer in East African Countries: A Policy Mapping Analysis

Diana Wangeshi Njuguna, Nour Mahrouseh, Dede Onisoyonivosekume and Orsolya Varga

Supplementary 1: List of Databases and Websites

Republic of Burundi: Available online: http://minisante.bi (accessed on 29 June 2019)
African Legal Information Institute: Available online: https://africanlii.org/liisearch?keyword=cancer (accessed on 04 March 2019)
International cancer control partnership: Available online: https://www.iccp-portal.org/ (accessed on 05 March 2019)
Country planning cycles Database (WHO): Available online: http://www.nationalplanningcycles.org (accessed on 05 March 2019)
Republic of Rwanda: Available online: http://www.moh.gov.rw/index.php?id=188 (accessed on 18 March 2019
Democratic Republic of Congo: Available online: http://sante.gouv.cd (accessed on 19 March 2019)
Union of the Comoros: Available online: https://www.gouvernement.km (accessed on 14 June 2019)
Republic of Kenya: Available online: http://www.health.go.ke (accessed on 25 March 2019)
Kenya Law: Available online: http://kenyalaw.org:8181/exist/kenyalex/index.xql (accessed on 25 March 2019)
Kenya Hospices and Palliative Care Association: Available online: http://kehpca.org/publications/ (accessed on 26 March 2019)
WHO Health law by countries: Available online: https://www.who.int/health-laws/countries/en/ (accessed on 28 March 2019)
Federal Democratic Republic of Ethiopia: Available online: http://www.moh.gov.et (accessed on 08 April 2019)
Ethiopia Law: Available online: https://www.abyssinialaw.com/ (accessed on 09 May 2019)
LexisNexis: Available online: https://lexisnexis.com (accessed on 09 April 2019)
Westlaw: Available online: https://legal.thomsonreuters.com/en/products/westlaw (accessed on 09 April 2019)
United Republic of Tanzania: Available online: http://www.tanzania.go.tz/ (accessed on 13 April 2019)
Ministry of Health Tanzania: Available online: http://www.mcdgc.go.tz/index.php/ (accessed on 07 May 2019)
Tanzania Legal Information Institute: Available online: https://tanzlii.org/ (accessed on 28 May 2019)

Republic of Madagascar: Available online: http://www.sante.gov.mg (accessed on 29 April 2019)

Republic of Uganda: Available online: https://health.go.ug/ (accessed on 13 May 2019)

Uganda Legal Information Institute: Available online: https://ulii.org/search/ulii/cancer (accessed on 11 March 2019)

Uganda Cancer Society: Available online: www.ugandacancersociety.org (accessed on 01 March 2019)

Uganda Cancer institute: Available online: https://www.uci.or.ug/ (accessed on 03 June 2019)

Federal Republic of Somalia: Available online: http://moh.gov.so/en/ (accessed on 24 April 2019).

Non-communicable Disease Document Repository. Available online: https://extranet.who.int/ncdccs/documents/db (accessed on 14 June 2019)

Supplementary 2: Prisma Flow Charts

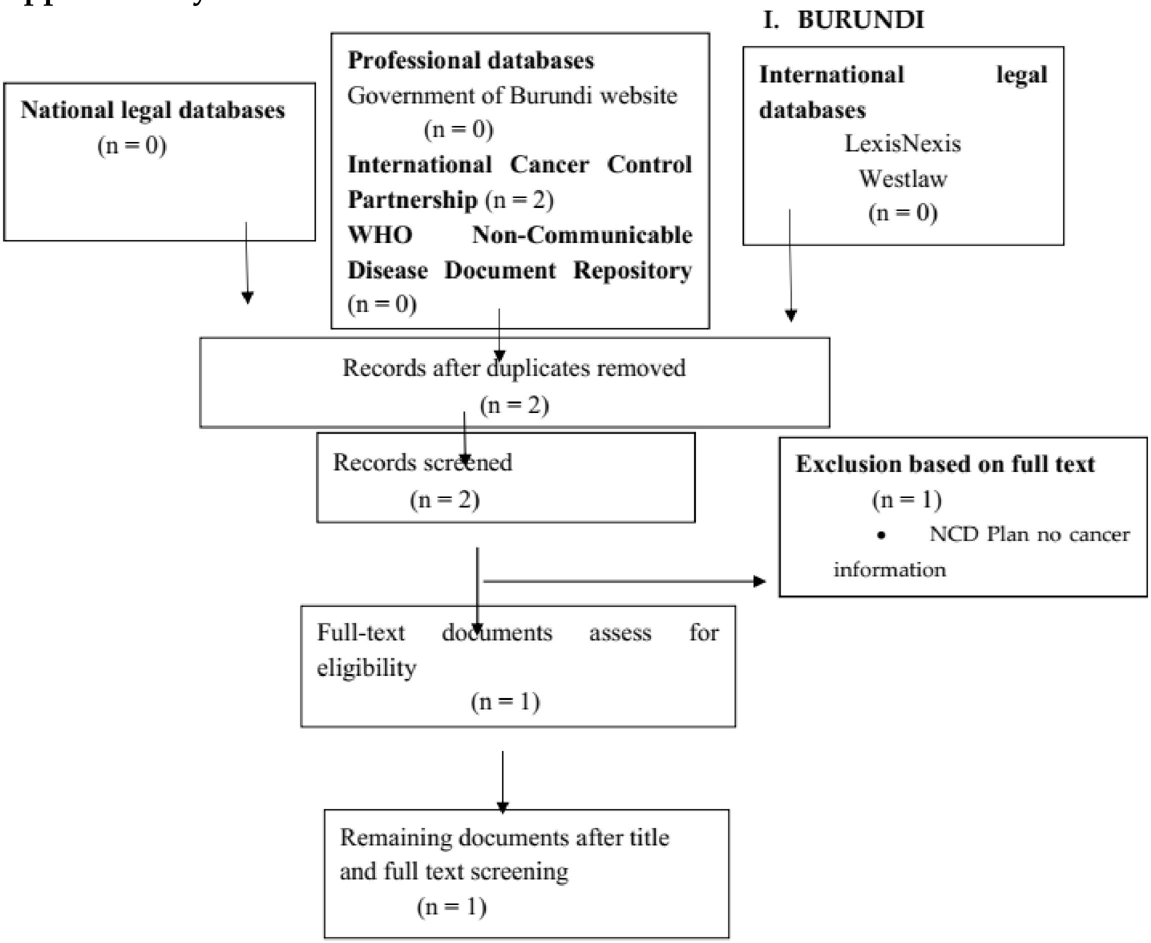

Figure S1. Selection of legal documents for Burundi

II. COMOROS

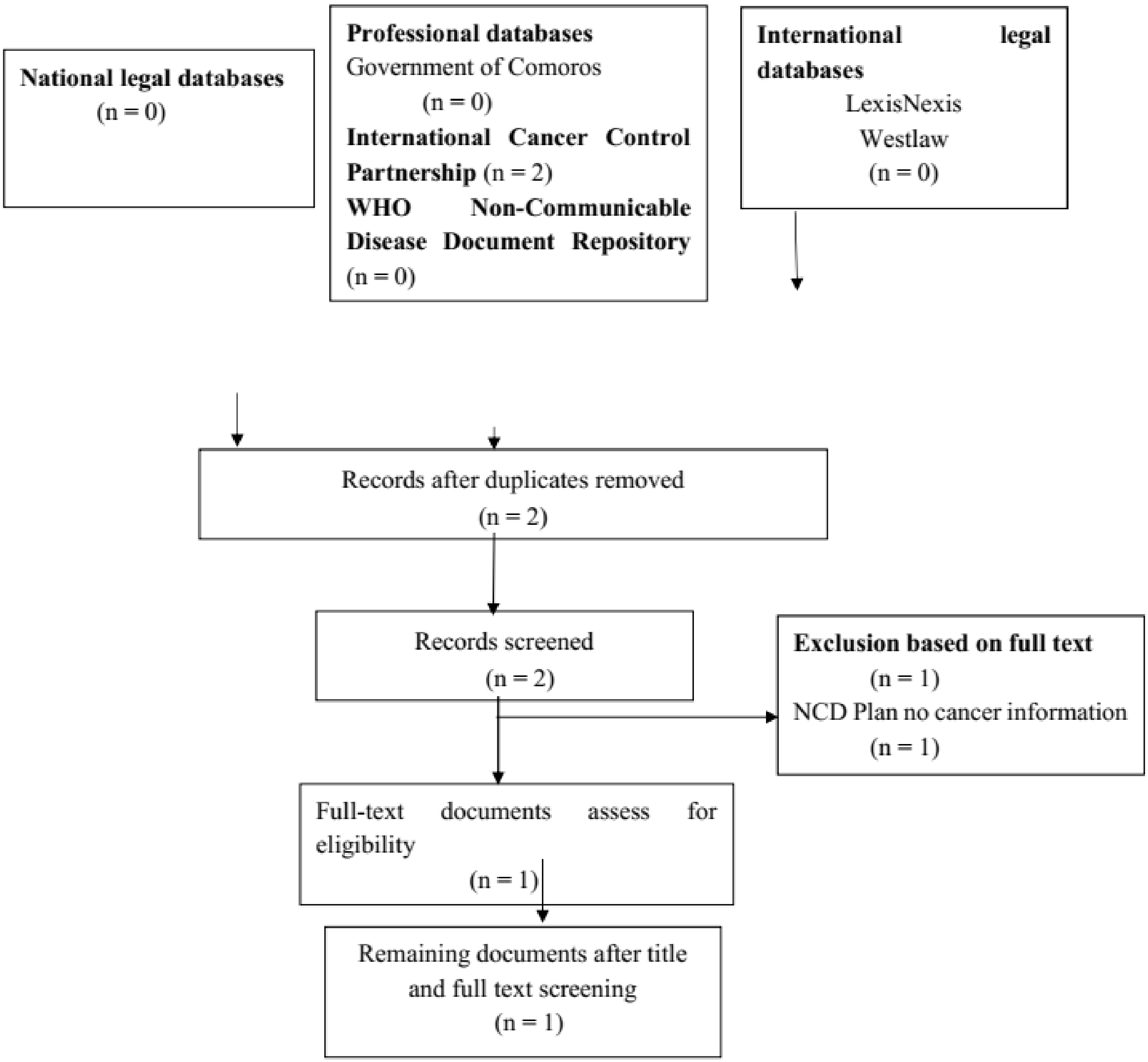

Figure S2. Selection of legal documents for Comoros

III. DJIBOUTI

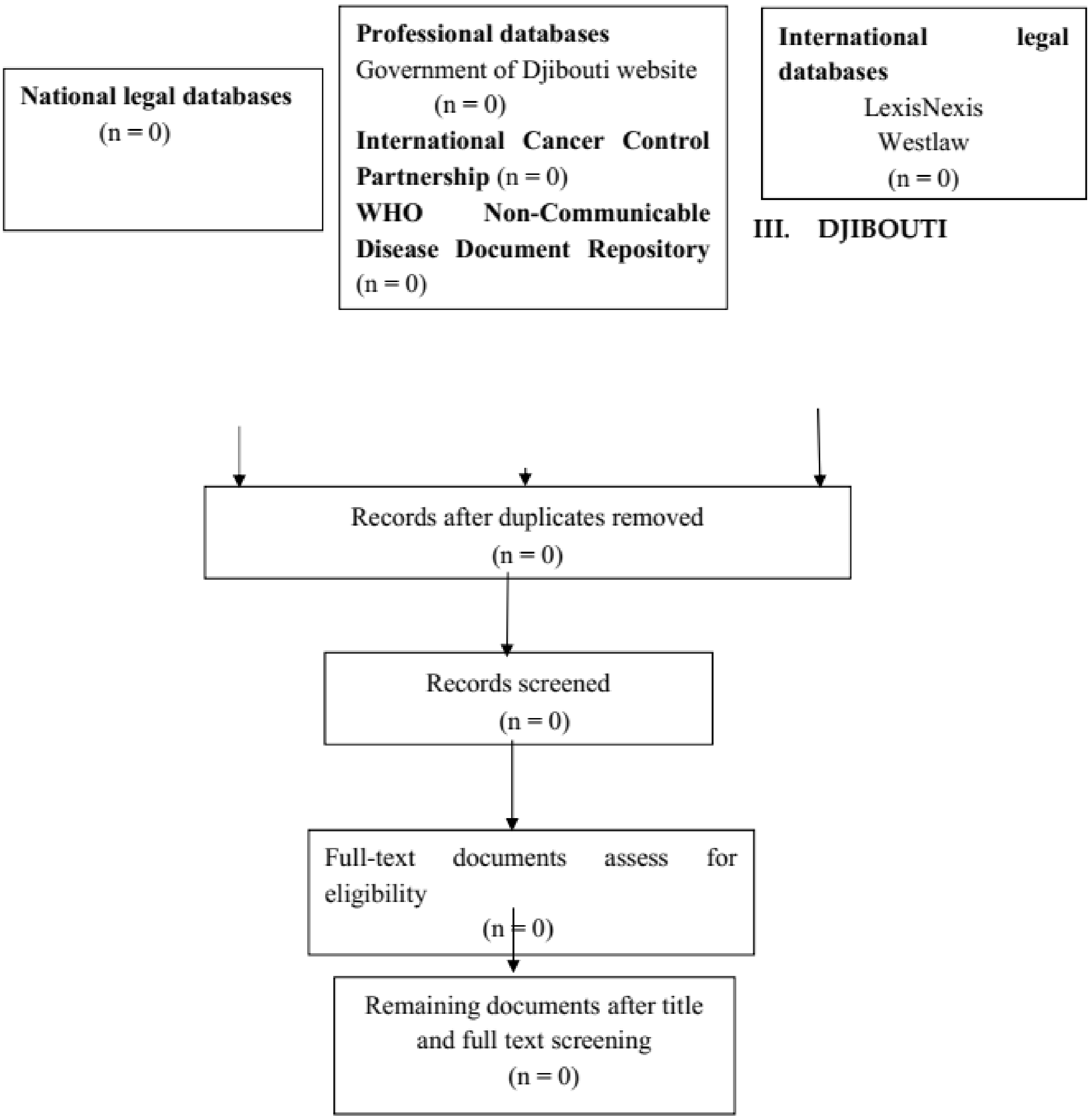

Figure S3. Selection of legal documents for Djibouti

IV. DEMOCRATIC REPUBLIC OF CONGO

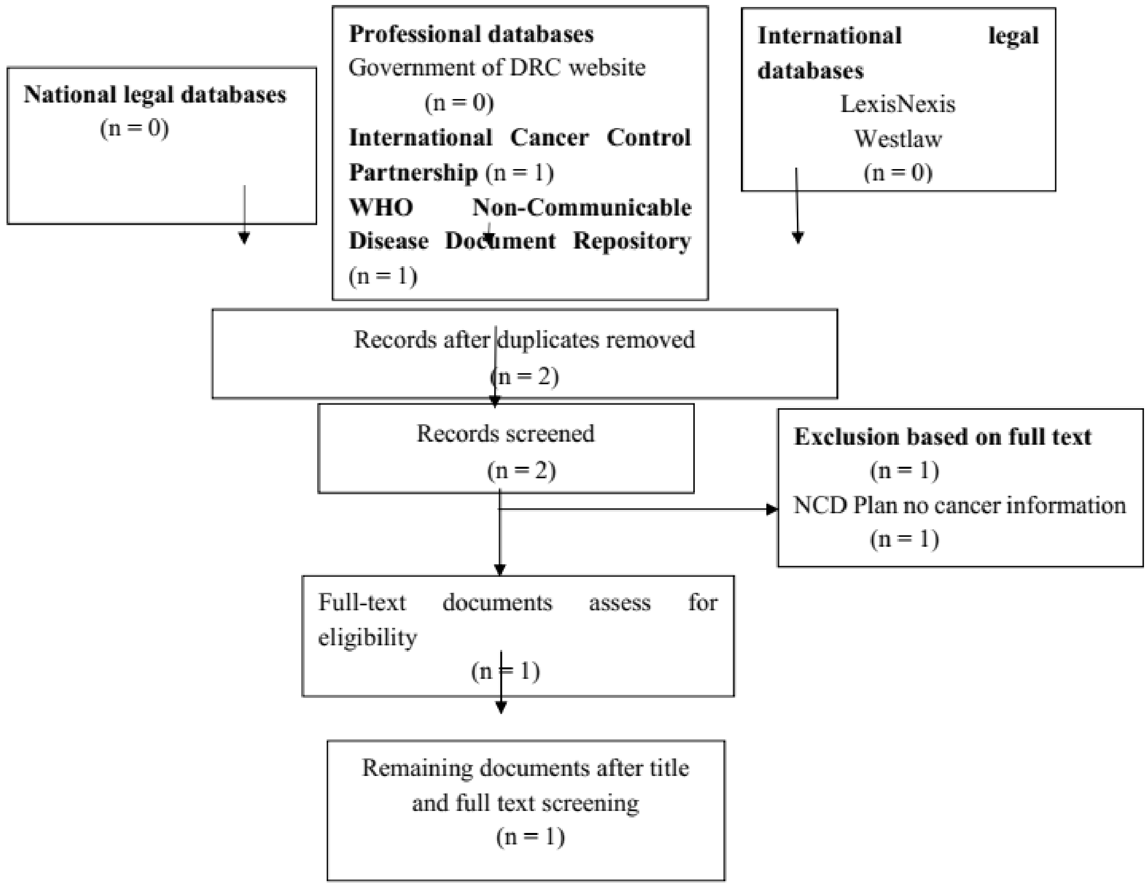

Figure S4. Selection of legal documents for Democratic Republic of Congo

V. ERITREA

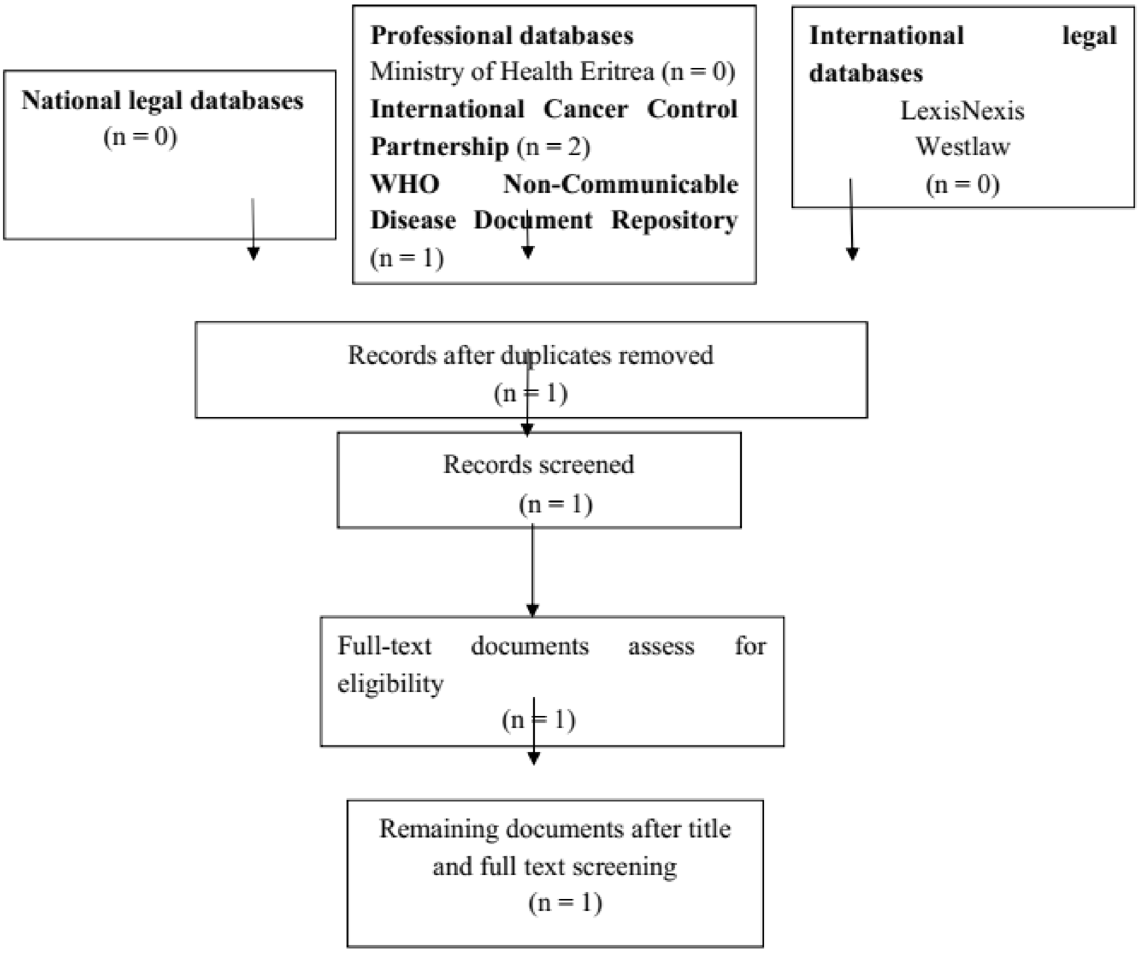

Figure S5. Selection of legal documents for Eritrea

VI. ETHIOPIA

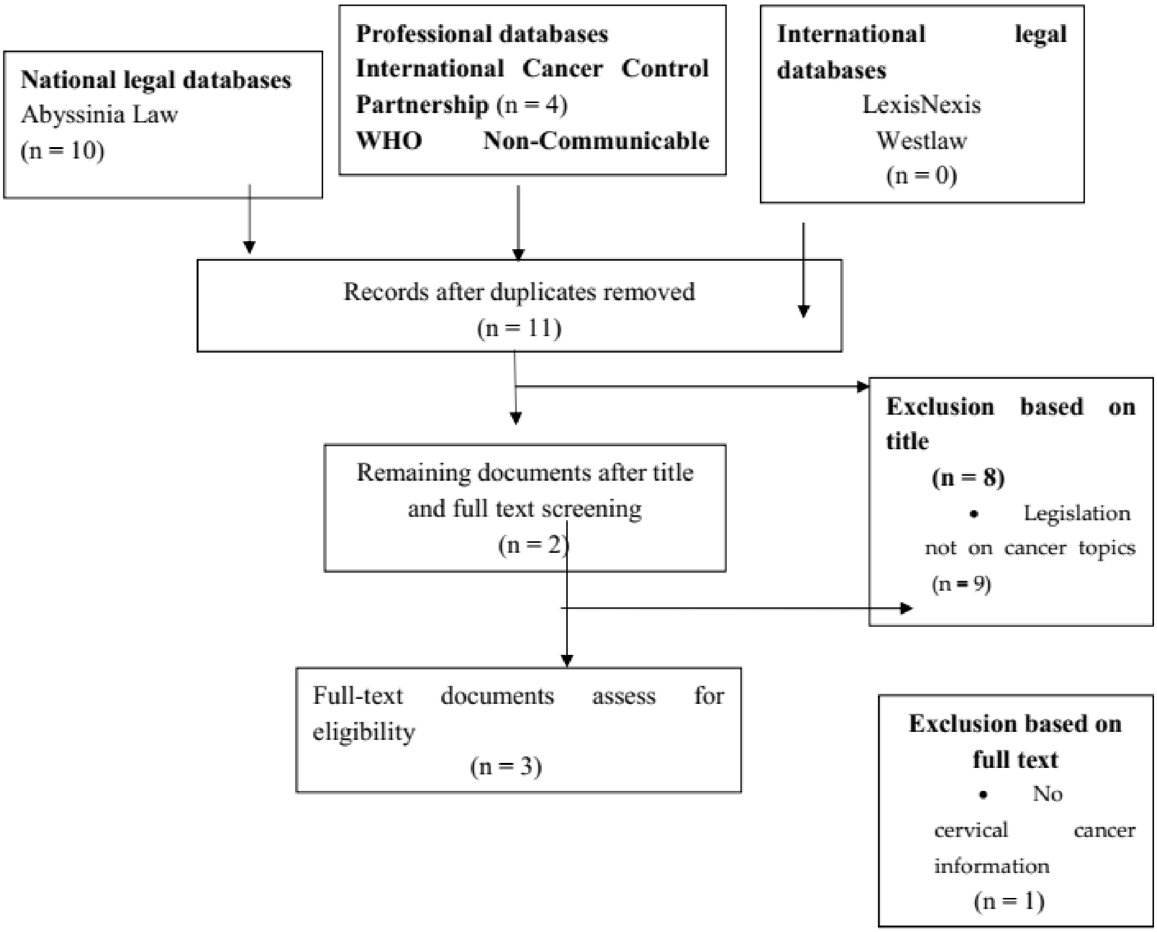

Figure S6. Selection of legal documents for Ethiopia

VII. KENYA

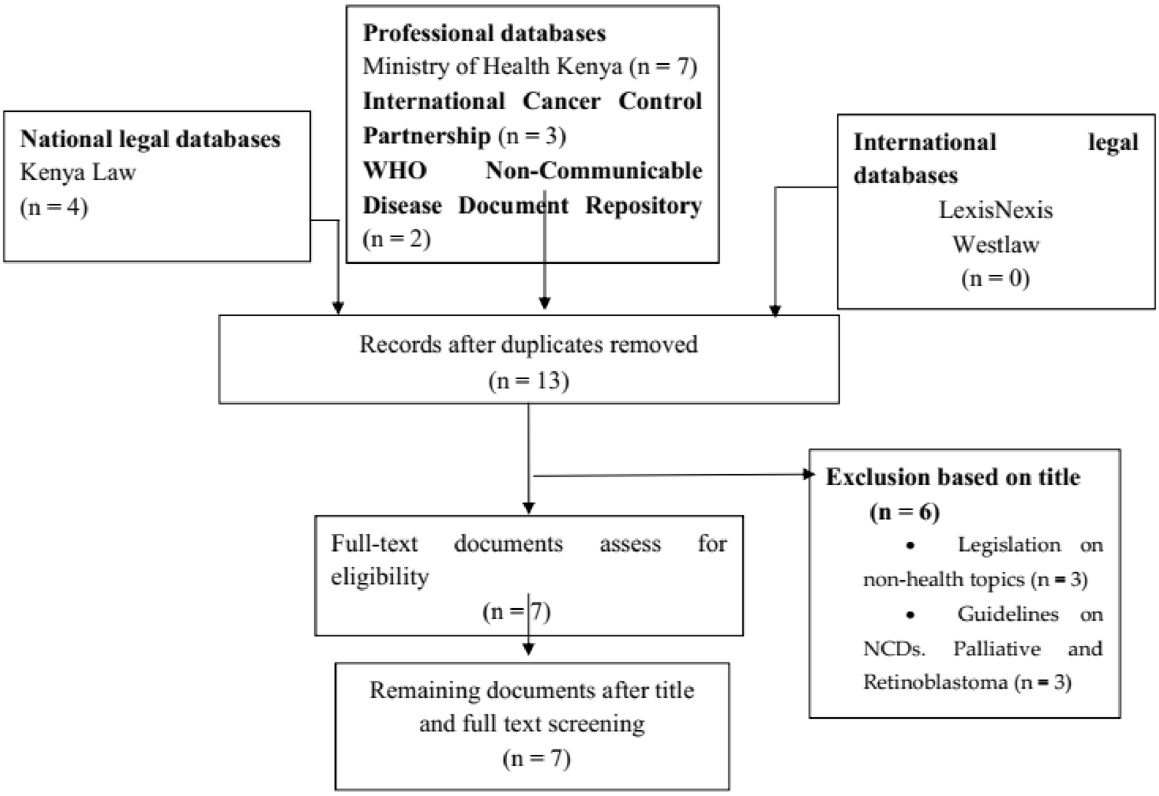

Figure S7. Selection of legal documents for Kenya

VIII. MADAGASCAR

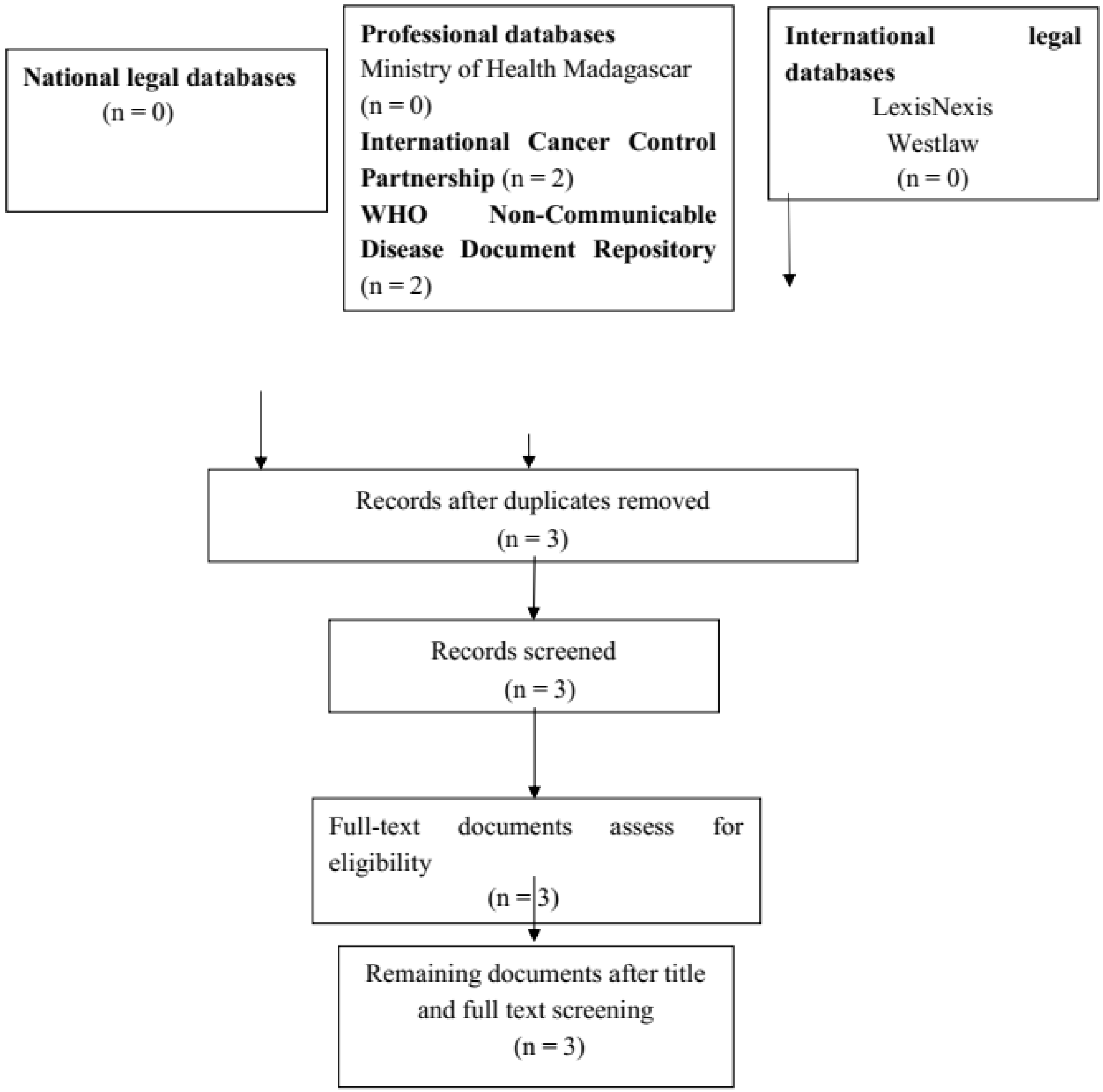

Figure S8. Selection of legal documents for Madagascar

IX. RWANDA

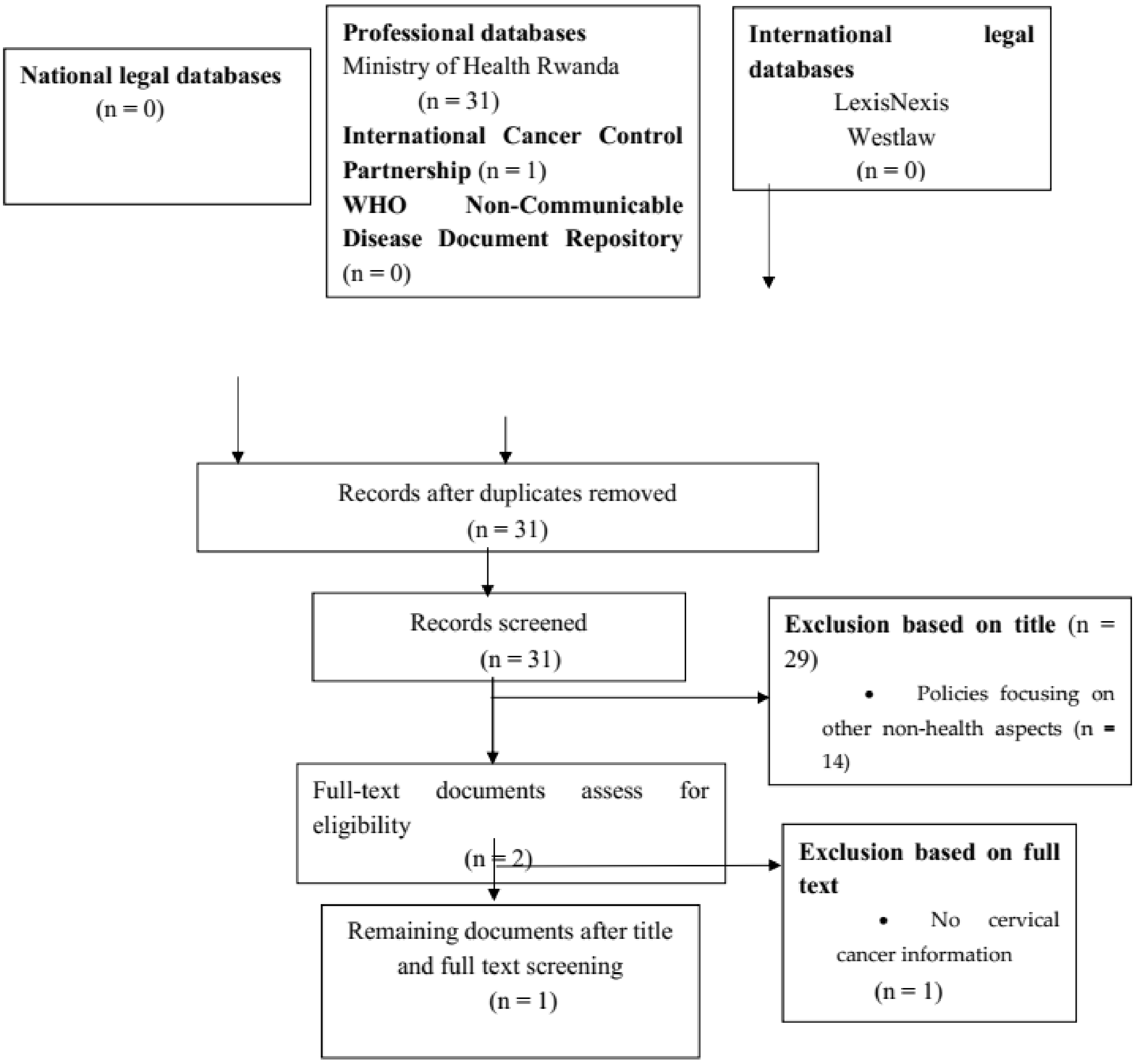

Figure S9. Selection of legal documents for Rwanda

X. SOMALIA

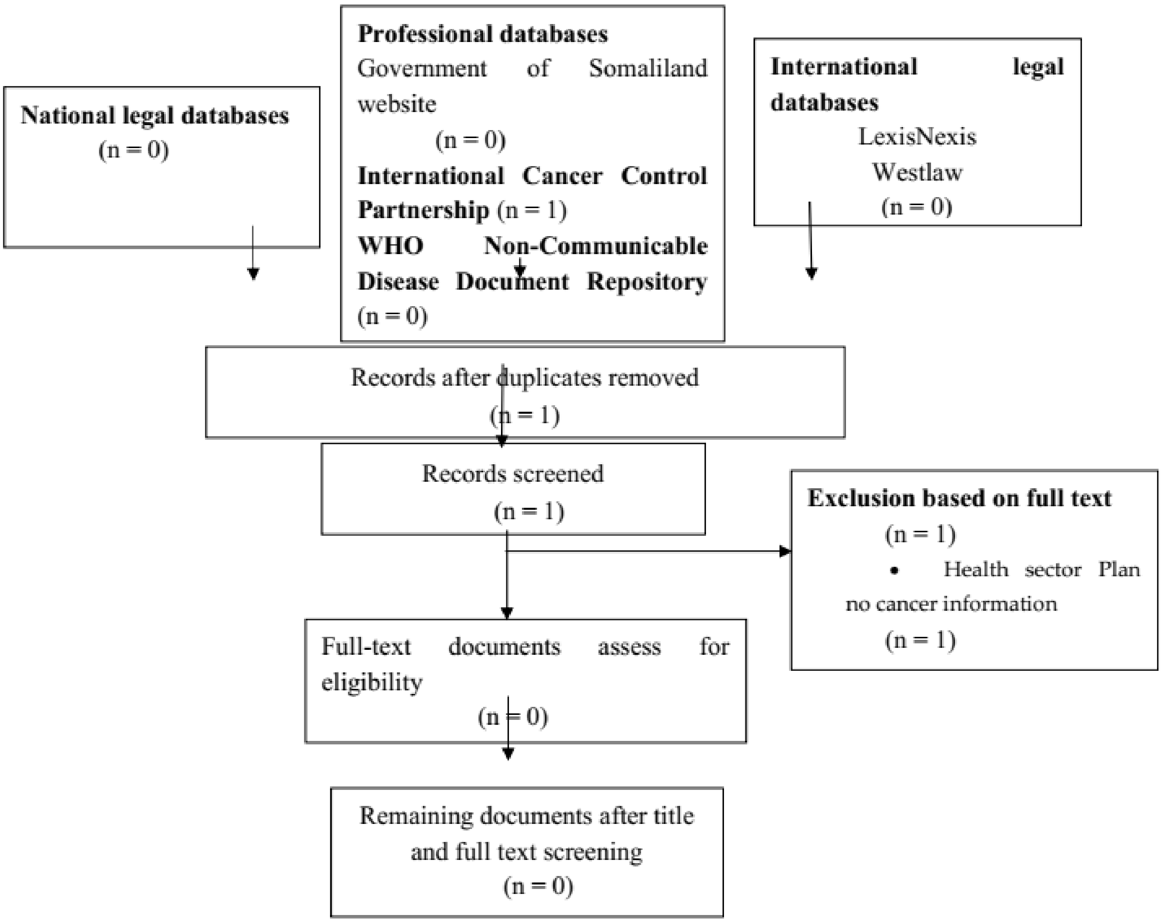

Figure S10. Selection of legal documents for Somalia

XI. TANZANIA

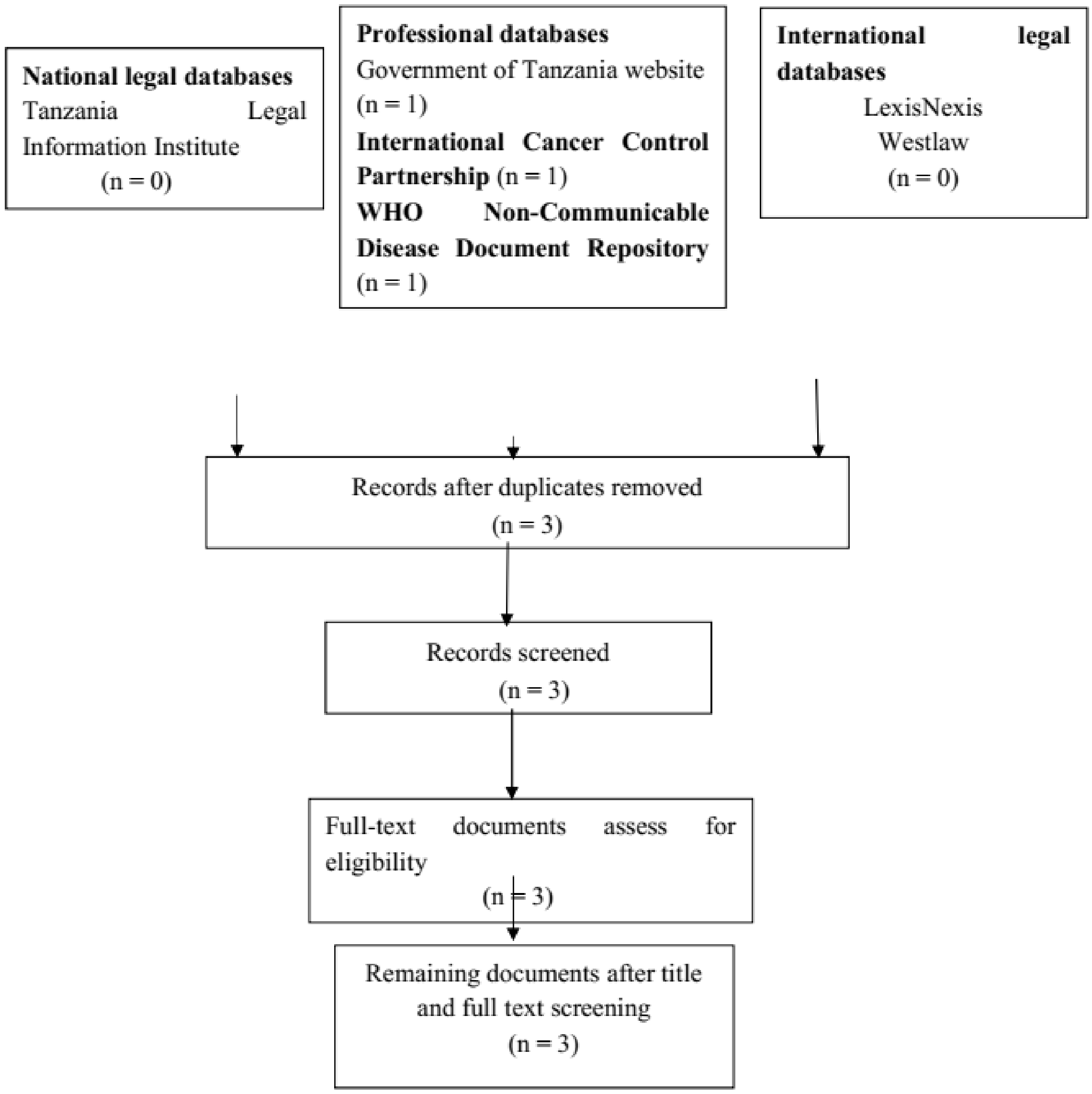

Figure S11. Selection of legal documents for Tanzania

XII. UGANDA

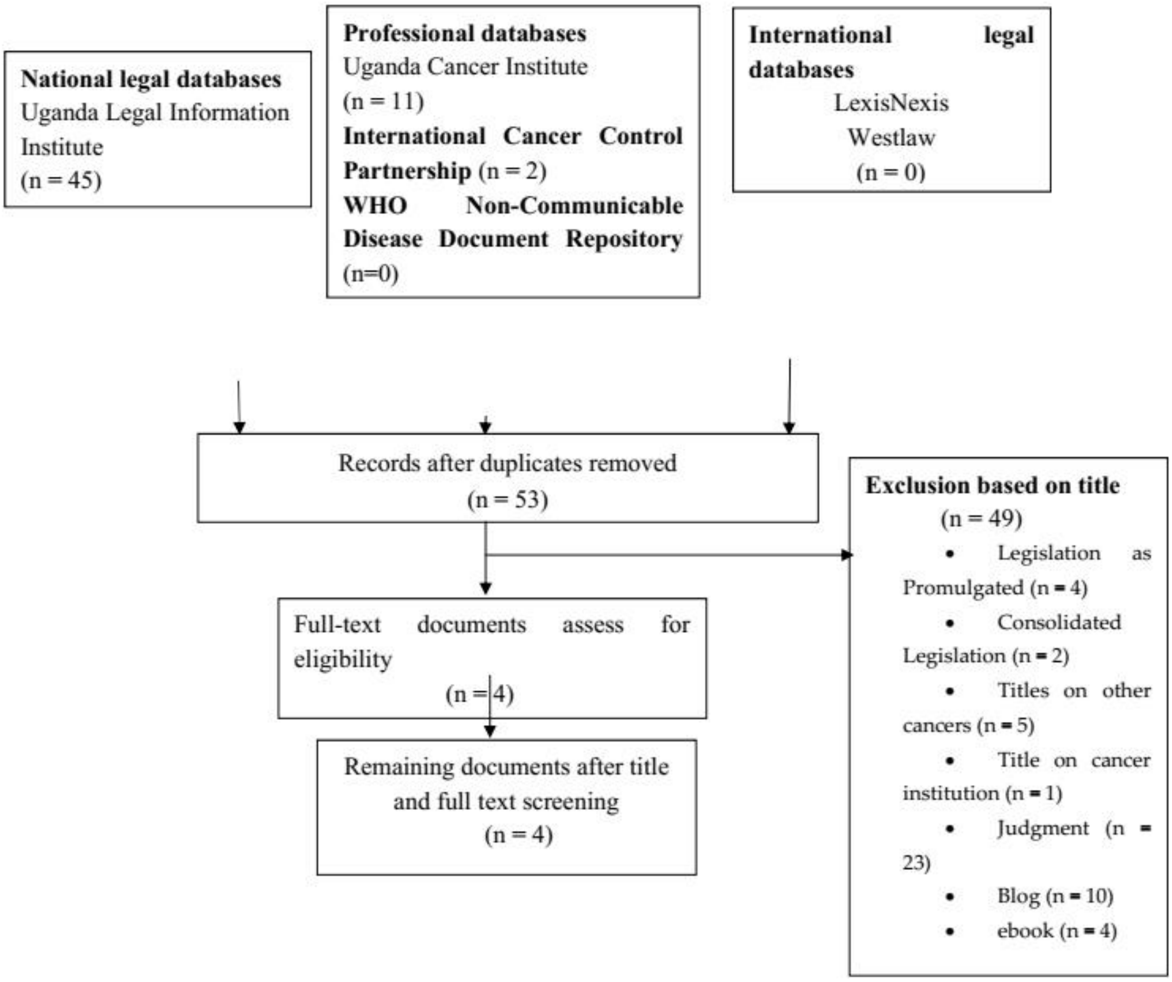

Figure S12. Selection of legal documents for Uganda

Supplementary 3: Internal Validity Assessment for the Included Legal Documents

Table S1. Internal validity assessment for the included legal documents, by domains.

**Countries**		**Kenya**							**Uganda**				**Tanzania**			**Ethiopia**		**Eritrea**	**Madagascar**			**Rwanda**	**Comoros**	**DRC**	**Burundi**
*Policy document Criteria		1	2	3	4	5	6	7	8	9	10	11	12	13	14	15	16	17	18	19	20	21	22	23	24
Background and case for change	Fulfilled	2	2		2	2		2	2	2			2	2		2				2	2			2	
	Room for improvement																1		1						1
	Not fulfilled			0			0				0	0			0			0				0	0		
Goals	Fulfilled	2			2	2				2						2	2				2				
	Room for improvement		1	1			1		1		1		1	1	1			1		1		1		1	
	Not fulfilled							0				0							0				0		0
	Fulfilled	2												2			2								

Resource considerations	Room for improvement		1	1	1	1		1	1	1	1		1		1	1					1			1	
	Not fulfilled						0					0						0	0	0		0	0		0
Monitoring and evaluation	Fulfilled	2			2			2	2	2			2	2		2	2				2				
	Room for improvement					1									1			1		1		1		1	
	Not fulfilled		0	0			0				0	0							0				0		0
Public opportunities	Fulfilled	2	2		2	2		2	2	2			2			2	2			2				2	
	Room for improvement										1			1				1	1		1	1	1		1
	Not fulfilled			0			0					0			0										
Obligations	Fulfilled	2			2	2				2				2			2				2				
	Room for improvement		1				1	1	1		1		1		1	1			1	1				1	1
	Not fulfilled			0								0						0				0	0		
	Fulfilled		2			2		2			2		2						2		2				

Potential for public health impact	Room for improvement	1			1				1	1				1	1	1	1			1			1	1	
	Not fulfilled			0			0					0						0				0			0
Total		13	9	2	12	12	2	10	10	12	6	0	11	11	5	11	12	3	5	8	12	3	2	9	3

Table S2. Internal validity assessment for the included legal documents, by domains, by Reviewer 1.

**Countries**		**Kenya**							**Uganda**				**Tanzania**			**Ethiopia**		**Eritrea**	**Madagascar**			**Rwanda**	**Comoros**	**DRC**	**Burundi**
*Policy document Criteria		1	2	3	4	5	6	7	8	9	10	11	12	13	14	15	16	17	18	19	20	21	22	23	24
Policy background	Fulfilled	2	2	0	2	2		2	2	2			2	2		2	2		2	2	2			2	
	Room for improvement																	1					1		1
	Not fulfilled				0		0				0	0			0							0			
Goals	Fulfilled	2			2	2			2	2			2	2		2	2							2	
	Room for improvement		1	1	0		1				1				1			1		1	1	1			
	Not fulfilled							0				0							0				0		0
Resources	Fulfilled	2											1				2								
	Room for improvement		1	1	1	1			1	1	1			1	1	1					1			1	
	Not fulfilled						0	1				0						0	0	0		0	0		0
Monitoring and evaluation	Fulfilled	2		0	2	2		2		2			2	2		2	2				2			1	
	Room for improvement								1											1		1			
	Not fulfilled		0		0		0				0	0			0			0	0				0		0
Public opportunities	Fulfilled	2	2		2	2		2	1	2			2	2		2	2			2				2	
	Room for improvement										1								1			1	1		1
	Not fulfilled			0			0					0			0			0			0				
Obligations	Fulfilled	2			2	2		1		2			2	2			2				2			2	
	Room for improvement			1			1		1		1				1	1									
	Not fulfilled		0									0						0	0	0		0	0		
Potential for public health impact	Fulfilled		2		2	2		2		2	2		2			2			2	2	2				0
	Room for improvement	1												1			1						1	1	
	Not fulfilled			0			0		0			0			0			0				0			0
Total		13	8	3	13	13	2	10	8	13	6	0	13	12	3	12	13	2	5	8	10	3	3	11	2

Table S3. Internal validity assessment for the included legal documents, by domains, by Reviewer 2.

**Countries**		**Kenya**							**Uganda**				**Tanzania**			**Ethiopia**		**Eritrea**	**Madagascar**			**Rwanda**	**Comoros**	**DRC**	**Burundi**
*Policy document Criteria		1	2	3	4	5	6	7	8	9	10	11	12	13	14	15	16	17	18	19	20	21	22	23	24
Background and case for change	Fulfilled	2	2		2	2		2	2	2			2			2				2	2			2	
	Room for improvement													1			1		1						1
	Not fulfilled			0			0				0	0			0			0				0	0		
Goals	Fulfilled	2			2	2				2						2	2				2				
	Room for improvement		1	1			1				1		1	1	1			1		1		1		1	
	Not fulfilled							0	0			0							0				0		0
Resource considerations	Fulfilled	2												2		2					2				
	Room for improvement		1	1	1	1		1	1	1	1		1		1		1							1	
	Not fulfilled						0					0						0	0	0		0	0		0
Monitoring and evaluation	Fulfilled				2			2	2				2	2		2					2				
	Room for improvement	1				1				1					1		1	1		1		1	1	1	
	Not fulfilled		0	0			0				0	0							0						0
Public opportunities	Fulfilled	2	2		2	2		2	2	2						2		2		2	2			2	
	Room for improvement			1							1		1	1	1		1		1			1	1		1
	Not fulfilled						0					0													
Obligations	Fulfilled				2	2				2						2	2			2	2				
	Room for improvement	1	1				1	1	1		1		1	1	1			1	1				1	1	1
	Not fulfilled			0								0										0			
	Fulfilled	2	2					2	2		2		2											2	

Potential for public health impact	Room for improvement				1	1								1	1	1	1		1	1	1				
	Not fulfilled			0			0			0		0						0				0	0		0
Total		12	9	3	12	11	2	10	10	10	6	0	10	9	6	13	9	5	4	9	13	3	3	10	3

References

1. Bray, F.; Ferlay, J.; Soerjomataram, I.; Siegel, R.L.; Torre, L.A.; Jemal, A. Global cancer statistics 2018: GLOBOCAN estimates of incidence and mortality worldwide for 36 cancers in 185 countries. *CA Cancer J Clin* **2018**, *68*, 394-424, doi:10.3322/caac.21492.
2. Ralaidovy, A.H.; Gopalappa, C.; Ilbawi, A.; Pretorius, C.; Lauer, J.A. Cost-effective interventions for breast cancer, cervical cancer, and colorectal cancer: new results from WHO-CHOICE. *Cost effectiveness and resource allocation : C/E* **2018**, *16*, 38, doi:10.1186/s12962-018-0157-0.
3. The Global Cancer Observatory. *Eastern Africa*; 2019.
4. Thrift-Perry, M.; Cabanes, A.; Cardoso, F.; Hunt, K.M.; Cruz, T.A.; Faircloth, K. Global analysis of metastatic breast cancer policy gaps and advocacy efforts across the patient journey. *The Breast* **2018**, *41*, 93-106, doi:10.1016/j.breast.2018.06.005.
5. World Health Organisation. Comprehensive cervical cancer control: a guide to essential practice; ISBN 978 92 4 154895 3 2014.
6. Jemal, A.; Bray, F.; Forman, D.; O'Brien, M.; Ferlay, J.; Center, M.; Parkin, D.M. Cancer burden in Africa and opportunities for prevention. **2012**, *118*, 4372-4384, doi:10.1002/cncr.27410.
7. Bongaarts, J. United Nations Statistics Division The World's Women 2015: Trends and Statistics New York: United Nations, 2015. 260 p. **2016**, *42*, 154-154, doi:10.1111/j.1728-4457.2016.00121.x.
8. Ngugi, C.W.; Boga, H.; Muigai, A.W.T.; Wanzala, P.; Mbithi, J.N. Factors Affecting Uptake of Cervical Cancer Early Detection Measures Among Women in Thika, Kenya. *Health Care for Women International* **2012**, *33*, 595-613, doi:10.1080/07399332.2011.646367.
9. Hasahya, O.T.; Berggren, V.; Sematimba, D.; Nabirye, R.C.; Kumakech, E. Beliefs, perceptions and health-seeking behaviours in relation to cervical cancer: a qualitative study among women in Uganda following completion of an HPV vaccination campaign. *Glob Health Action* **2016**, *9*, 29336, doi:10.3402/gha.v9.29336.
10. Marks-Sultan, G.; Tsai, F.J.; Anderson, E.; Kastler, F.; Sprumont, D.; Burris, S. National public health law: a role for WHO in capacity-building and promoting transparency. *Bull World Health Organ* **2016**, *94*, 534-539, doi:10.2471/blt.15.164749.
11. Burris, S.C. Building the Discipline of Policy Surveillance: Report and Next Steps from an International Convening; Center for Public Health Law Research, Temple University, 18th January 2018, 2018.
12. World Health Organization. *Strategizing National Health in the 21st century: A handbook*; World Health Organization: Geneva, 2016.
13. Public Health Law Centre. Laws, Policies and Regulations: Key Terms & Concepts; 15/03/2020, 2015.
14. United States Government. Introduction to United States Law & Policy. 2018.
15. Johanson, J.-E.; Pekkola, E.; Husman, P. Government Programme as a Strategy—The Finnish Experience. *Administrative Sciences* **2017**, *7*, 16.
16. Espina, C.; Soerjomataram, I.; Forman, D.; Martín-Moreno, J.M. Cancer prevention policy in the EU: Best practices are now well recognised; no reason for countries to lag behind. *Journal of Cancer Policy* **2018**, *18*, 40-51, doi:https://doi.org/10.1016/j.jcpo.2018.09.001.
17. World Health Organization. National Cancer Control Programmes: Policies and Managerial Guidelines; World Health Organization: 2002.
18. Hong, G.; White, J.; Zhong, L.; Carlson, L.E. Survey of Policies and Guidelines on Antioxidant Use for Cancer Prevention, Treatment, and Survivorship in North American Cancer Centers: What Do Institutions Perceive as Evidence? *Integrative Cancer Therapies* **2015**, *14*, 305-317, doi:10.1177/1534735415572884.
19. Callahan, R.; Darzi, A. Five Policy Levers To Meet The Value Challenge In Cancer Care. *Health Affairs* **2015**, *34*, 1563-1568, doi:10.1377/hlthaff.2015.0308.
20. Petry, K.U.; Wörmann, B.; Schneider, A. Benefits and Risks of Cervical Cancer Screening. *Oncology Research and Treatment* **2014**, *37(suppl 3)*, 48-57, doi:10.1159/000365059.
21. Ginsberg, G.M.; Lauer, J.A.; Zelle, S.; Baeten, S.; Baltussen, R. Cost effectiveness of strategies to combat breast, cervical, and colorectal cancer in sub-Saharan Africa and South East Asia: mathematical modelling study. *BMJ (Clinical research ed.)* **2012**, *344*, e614, doi:10.1136/bmj.e614.
22. Ginsburg, O.M. Breast and cervical cancer control in low and middle-income countries: Human rights meet sound health policy. *Journal of Cancer Policy* **2013**, *1*, e35-e41, doi:https://doi.org/10.1016/j.jcpo.2013.07.002.
23. Duggan, C.; Coronado, G.; Martinez, J.; Byrd, T.L.; Carosso, E.; Lopez, C.; Benavides, M.; Thompson, B. Cervical cancer screening and adherence to follow-up among Hispanic women study protocol: a

randomized controlled trial to increase the uptake of cervical cancer screening in Hispanic women. *BMC Cancer* **2012**, *12*, 170-170, doi:10.1186/1471-2407-12-170.
24. Saslow, D.; Solomon, D.; Lawson, H.W.; Killackey, M.; Kulasingam, S.L.; Cain, J.; Garcia, F.A.R.; Moriarty, A.T.; Waxman, A.G.; Wilbur, D.C., et al. American Cancer Society, American Society for Colposcopy and Cervical Pathology, and American Society for Clinical Pathology screening guidelines for the prevention and early detection of cervical cancer. *CA Cancer J Clin* **2012**, *62*, 147-172, doi:10.3322/caac.21139.
25. World Health Organisation. Cancer Control: Knowledge into Action: WHO Guide for Effective Programmes. **2007**.
26. Maine, D.; Hurlburt, S.; Greeson, D. Cervical cancer prevention in the 21st century: cost is not the only issue. *Am J Public Health* **2011**, *101*, 1549-1555, doi:10.2105/AJPH.2011.300204.
27. Ghosh, P.; Gandhi, G.; Kochhar, P.K.; Zutshi, V.; Batra, S. Visual inspection of cervix with Lugol's iodine for early detection of premalignant & malignant lesions of cervix. *Indian J Med Res* **2012**, *136*, 265-271.
28. Denny, L.; Kuhn, L.; De Souza, M.; Pollack, A.E.; Dupree, W.; Wright, T.C. Screen-and-Treat Approaches for Cervical Cancer Prevention in Low-Resource SettingsA Randomized Controlled Trial. *JAMA* **2005**, *294*, 2173-2181, doi:10.1001/jama.294.17.2173 %J JAMA.
29. United Nations. *World Economic Situation Prospects*; United Nations publication
30. 2013.
31. World Atlas. Complete List Of Country Abbreviations. Availabe online: https://www.worldatlas.com/aatlas/ctycodes.htm (accessed on 18 December 2019).
32. World Health Organization. *National cancer control programmes : policies and managerial guidelines*, 2nd Edition ed.; World Health Organization
33. Geneva, Switzerland, 2002.
34. Cheung, K.K.; Mirzaei, M.; Leeder, S. Health policy analysis: a tool to evaluate in policy documents the alignment between policy statements and intended outcomes. *Australian health review : a publication of the Australian Hospital Association* **2010**, *34*, 405-413, doi:10.1071/ah09767.
35. Briggs, A.M.; Persaud, J.G.; Deverell, M.L.; Bunzli, S.; Tampin, B.; Sumi, Y.; Amundsen, O.; Houlding, E.M.G.; Cardone, A.; Hugosdottir, T., et al. Integrated prevention and management of non-communicable diseases, including musculoskeletal health: a systematic policy analysis among OECD countries. *BMJ Global Health* **2019**, *4*, e001806, doi:10.1136/bmjgh-2019-001806.
36. Fleiss, J.L.; Cohen, J. The Equivalence of Weighted Kappa and the Intraclass Correlation Coefficient as Measures of Reliability. **1973**, *33*, 613-619, doi:10.1177/001316447303300309.
37. Cohen, J. A Coefficient of Agreement for Nominal Scales. **1960**, *20*, 37-46, doi:10.1177/001316446002000104.
38. Republic of Burundi. Politique Nationale Sante 2016-2025. 2016.
39. Democratic Republic of Congo. National strategy to combat cancer of the uterine neck and breast in Democratic Republic of Congo. 2015.
40. State of Eritrea. Health Sector Strategic Development plan. 2016.
41. Federal Democratic Republic of Ethiopia. Guideline for cervical cancer prevention and control. 2015.
42. Federal Democratic Republic of Ethiopia. National Cancer Control Plan. 2015.
43. Union of the Comoros. Cadre Politique Reproduction. 2002.
44. Republic of Madagascar. National Strategic Plan to fight against cervical cancer 2016.
45. Republic of Madagascar. National Cancer Policy of Madagascar 2010.
46. Republic of Madagascar. Cervical Cancer Screening Guide fr.en.
47. Uganda Cancer Society. Uganda Cancer Society Strategic Plan 2016.
48. Republic of Uganda. Strategic plan for Cervical cancer prevention and control in Uganda. 2010.
49. Republic of Uganda. Cancer Institute Act. 2017.
50. Uganda Cancer Institute. Uganda Cance Institute Treatment Guidelines. 2017.
51. United Republic of Tanzania. The Ocean Road Cancer Institute Act. 1996.
52. United Republic of Tanzania. National cancer control strategy. 2013.
53. United Republic of Tanzania. National Cervical Cancer Prevention and control strategic plan 2011.
54. Republic of Rwanda. Health Sector Strategic Plan. 2018.
55. Republic of Kenya. Cancer Prevention and Control Amendment Bill 2017.
56. Republic of Kenya. Cancer Prevention and Control Act 2012.
57. Republic of Kenya. Kenya National Cervical Cancer Prevention Plan 2012.

58. Republic of Kenya. National Guidelines for Prevention and Management of Cervical, Breast and Prostate Cancers. 2012.
59. Republic of Kenya. National Cancer Treatment Guidelines. 2013.
60. Republic of Kenya. National Cancer Control strategy 2011- 2016. 2011.
61. Republic of Kenya. National Cancer Control Strategy 2017-2022. 2017.
62. Finocchario-Kessler, S.; Wexler, C.; Maloba, M.; Mabachi, N.; Ndikum-Moffor, F.; Bukusi, E. Cervical cancer prevention and treatment research in Africa: a systematic review from a public health perspective. *BMC Womens Health* **2016**, *16*, 29-29, doi:10.1186/s12905-016-0306-6.
63. Huchko, M.J.; Sneden, J.; Leslie, H.H.; Abdulrahim, N.; Maloba, M.; Bukusi, E.; Cohen, C.R. A comparison of two visual inspection methods for cervical cancer screening among HIV-infected women in Kenya. *Bulletin of the World Health Organization* **2014**, *92*, 195-203, doi:10.2471/BLT.13.122051.
64. Sankaranarayanan, R. Screening for Cancer in Low- and Middle-Income Countries. *Annals of Global Health* **2014**, *80*, 412-417, doi:https://doi.org/10.1016/j.aogh.2014.09.014.
65. World Health Organization, I.A.f.R.o.C., African Population Health Research Center, . *Prevention of cervical cancer through screening using visual inspection with acetic acid (VIA) and treatment with cryotherapy.* ; World Health Organization: Geneva, 2012.
66. Catarino, R.; Petignat, P.; Dongui, G.; Vassilakos, P. Cervical cancer screening in developing countries at a crossroad: Emerging technologies and policy choices. *World J Clin Oncol* **2015**, *6*, 281-290, doi:10.5306/wjco.v6.i6.281.
67. Chibwesha, C.J.; Cu-Uvin, S. See-and-treat approaches to cervical cancer prevention for HIV-infected women. *Curr HIV/AIDS Rep* **2011**, *8*, 192-199, doi:10.1007/s11904-011-0084-6.
68. Lince-Deroche, N.; Phiri, J.; Michelow, P.; Smith, J.S.; Firnhaber, C. Costs and Cost Effectiveness of Three Approaches for Cervical Cancer Screening among HIV-Positive Women in Johannesburg, South Africa. *PLOS ONE* **2015**, *10*, e0141969, doi:10.1371/journal.pone.0141969.
69. Malloy, C.; Sherris, J.; Herdman, C. HPV DNA Testing: Technical and Programmatic. Seattle, Washington, Program for Appropriate Technology in Health [*http://www*. popline. org] **2000**.
70. Kuhn, L.; Denny, L.; Pollack, A.; Lorincz, A.; Richart, R.M.; Wright, T.C. Human Papillomavirus DNA Testing for Cervical Cancer Screening in Low-Resource Settings. *JNCI: Journal of the National Cancer Institute* **2000**, *92*, 818-825, doi:10.1093/jnci/92.10.818.
71. Republic of Uganda. Cervical cancer strategic plan 2010-2014. 2010.
72. Mboumba Bouassa, R.S.; Prazuck, T.; Lethu, T.; Jenabian, M.A.; Meye, J.F.; Belec, L. Cervical cancer in sub-Saharan Africa: a preventable noncommunicable disease. *Expert review of anti-infective therapy* **2017**, *15*, 613-627, doi:10.1080/14787210.2017.1322902.
73. World Health Organization. Guide to introducing HPV vaccine into national immunization programmes. **2016**.
74. Patel, C.; Brotherton, J.M.; Pillsbury, A.; Jayasinghe, S.; Donovan, B.; Macartney, K.; Marshall, H. The impact of 10 years of human papillomavirus (HPV) vaccination in Australia: what additional disease burden will a nonavalent vaccine prevent? *Euro Surveill* **2018**, *23*, doi:10.2807/1560-7917.ES.2018.23.41.1700737.
75. Nwogu, C.; Mahoney, M.; George, S.; Dy, G.; Hartman, H.; Animashaun, M.; Popoola, A.; Michalek, A. Promoting cancer control training in resource limited environments: Lagos, Nigeria. *J Cancer Educ* **2014**, *29*, 14-18, doi:10.1007/s13187-013-0581-y.
76. Tchounga, B.K.; Jaquet, A.; Coffie, P.A.; Horo, A.; Sauvaget, C.; Adoubi, I.; Guie, P.; Dabis, F.; Sasco, A.J.; Ekouevi, D.K. Cervical cancer prevention in reproductive health services: knowledge, attitudes and practices of midwives in Côte d'Ivoire, West Africa. *BMC Health Serv Res* **2014**, *14*, 165-165, doi:10.1186/1472-6963-14-165.
77. Jemal, A.; Brawley, O.W. Increasing cancer awareness and prevention in Africa. *Ecancermedicalscience* **2019**, *13*, 939-939, doi:10.3332/ecancer.2019.939.
78. Black, E.; Richmond, R. Prevention of Cervical Cancer in Sub-Saharan Africa: The Advantages and Challenges of HPV Vaccination. *Vaccines (Basel)* **2018**, *6*, 61, doi:10.3390/vaccines6030061.
79. Di, J.; Rutherford, S.; Chu, C. Review of the Cervical Cancer Burden and Population-Based Cervical Cancer Screening in China. *Asian Pac J Cancer Prev* **2015**, *16*, 7401-7407, doi:10.7314/apjcp.2015.16.17.7401.
80. Vu, M.; Yu, J.; Awolude, O.A.; Chuang, L. Cervical cancer worldwide. *Current Problems in Cancer* **2018**, *42*, 457-465, doi:https://doi.org/10.1016/j.currproblcancer.2018.06.003.

81. Anorlu, R.I. Cervical cancer: the sub-Saharan African perspective. *Reproductive Health Matters* **2008**, *16*, 41-49, doi:10.1016/S0968-8080(08)32415-X.
82. Nkfusai, N.C.; Cumber, S.N.; Williams, T.; Anchang-Kimbi, J.K.; Yankam, B.M.; Anye, C.S.; Tsoka-Gwegweni, J.M.; George Enow, E.-O.; Anong, D.N. Cervical cancer in the Bamenda Regional Hospital, North West Region of Cameroon: a retrospective study. *Pan Afr Med J* **2019**, *32*, 90-90, doi:10.11604/pamj.2019.32.90.18217.
83. Gatumo, M.; Gacheri, S.; Sayed, A.-R.; Scheibe, A. Women's knowledge and attitudes related to cervical cancer and cervical cancer screening in Isiolo and Tharaka Nithi counties, Kenya: a cross-sectional study. *BMC Cancer* **2018**, *18*, 745-745, doi:10.1186/s12885-018-4642-9.
84. Tefera, F.; Mitiku, I. Uptake of Cervical Cancer Screening and Associated Factors Among 15–49-Year-Old Women in Dessie Town, Northeast Ethiopia. *Journal of Cancer Education* **2017**, *32*, 901-907, doi:10.1007/s13187-016-1021-6.
85. Shiferaw, S.; Addissie, A.; Gizaw, M.; Hirpa, S.; Ayele, W.; Getachew, S.; Kantelhardt, E.J.; Assefa, M.; Jemal, A. Knowledge about cervical cancer and barriers toward cervical cancer screening among HIV-positive women attending public health centers in Addis Ababa city, Ethiopia. *Cancer Med* **2018**, *7*, 903-912, doi:10.1002/cam4.1334.
86. Geertsen, M.; Bais, A.G.; Beerman, H.; Helmerhorst, T.J. [Follow up after an abnormal pap smear: time interval acceptable, nature of follow up leaves room for improvement]. *Ned Tijdschr Geneeskd* **2003**, *147*, 2430-2434.
87. Nartey, Y.; Hill, P.; Amo-Antwi, K.; Asmah, R.; Nyarko, K.; Yarney, J.; Damale, N.; Cox, B. Recommendations for cervical cancer prevention and control in Ghana: public education and human papillomavirus vaccination. *Ghana Med J* **2018**, *52*, 94-102, doi:10.4314/gmj.v52i2.6.
88. Morhason-Bello, I.O.; Odedina, F.; Rebbeck, T.R.; Harford, J.; Dangou, J.-M.; Denny, L.; Adewole, I.F. Challenges and opportunities in cancer control in Africa: a perspective from the African Organisation for Research and Training in Cancer. *The lancet oncology* **2013**, *14*, e142-e151.
89. Malekinejad, M.; Horvath, H.; Snyder, H.; Brindis, C.D. The discordance between evidence and health policy in the United States: the science of translational research and the critical role of diverse stakeholders. *Health Research Policy and Systems* **2018**, *16*, 81, doi:10.1186/s12961-018-0336-7.
90. Fadlelmola, F.M. Cancer registries and cancer genomics research in east africa: challenges and lessons learned. *International Clinical Pathology Journal* **2016**, *2*, 67–76, doi:10.15406/icpjl.2016.02.00045.
91. White, M.C.; Babcock, F.; Hayes, N.S.; Mariotto, A.B.; Wong, F.L.; Kohler, B.A.; Weir, H.K. The history and use of cancer registry data by public health cancer control programs in the United States. *Cancer* **2017**, *123 Suppl 24*, 4969-4976, doi:10.1002/cncr.30905.
92. Martei, Y.M.; Pace, L.E.; Brock, J.E.; Shulman, L.N. Breast Cancer in Low- and Middle-Income Countries: Why We Need Pathology Capability to Solve This Challenge. *Clin Lab Med* **2018**, *38*, 161-173, doi:10.1016/j.cll.2017.10.013.
93. Black, E.; Richmond, R. Improving early detection of breast cancer in sub-Saharan Africa: why mammography may not be the way forward. *Global Health* **2019**, *15*, 3, doi:10.1186/s12992-018-0446-6.
94. de-Graft Aikins, A.; Unwin, N.; Agyemang, C.; Allotey, P.; Campbell, C.; Arhinful, D. Tackling Africa's chronic disease burden: from the local to the global. *Global Health* **2010**, *6*, 5-5, doi:10.1186/1744-8603-6-5.
95. Andermann, A.; Pang, T.; Newton, J.N.; Davis, A.; Panisset, U. Evidence for Health II: Overcoming barriers to using evidence in policy and practice. *Health Research Policy and Systems* **2016**, *14*, 17, doi:10.1186/s12961-016-0086-3.
